# Effect of grain shape elongation in direction and perpendicular to current stream (conductive or insulator) on the electrical characteristics of mixtures

**DOI:** 10.1038/s41598-024-83223-8

**Published:** 2025-01-10

**Authors:** Mohamed Mahmoud Gomaa

**Affiliations:** National Research Centre, Geophysical Sciences Department, Geophysical Exploration Lab., Dokki, Cairo, Egypt

**Keywords:** Grain shape, Current stream, Conductivity, Dielectric constant, Mixtures, Effective medium, Solid Earth sciences, Geophysics

## Abstract

The shape elongation of grains, in mixtures, can have an effect on the electrical characteristics. Grain size and structural changes can impact the dielectric, and electrical properties of materials. The electrical properties of natural mixtures are influenced by their arrangement and shape. Insulating grains block potential pathways for electricity between electrodes. One key factor is grain shape elongation and how it aligns with the electric current. If the long part of the conductor grains lines up with the stream of current, then it will show high conductivity and an early critical percolation threshold. The critical percolation threshold decreases when the conductor is extended with the stream of current. Conductor elongated with the current stream increases the current flow. When the conductor is elongated perpendicular to the current stream, from sphere to needle, no perceptible alterations upon the associated critical threshold take effect. When the insulator is elongated with the stream of current the critical percolation threshold does not change significantly. The critical percolation threshold increases when insulator elongation perpendicular to the current direction. The elongated insulator perpendicular to the current’s stream hinders the current’s flow. The EMT offers a simulation to contain the variations in dielectric constant and conductivity across different concentrations. This is the first time to discuss the direction of elongation of conductor or insulator grains, in a mixture, in parallel or perpendicular to the stream of the current.

## Introduction

The electrical behavior of rocks is influenced by the different grains and cements that make up the rock, as well as the shape and orientation of the grains^1^. The electrical conductivity of rocks can be affected by fluid conduction, rock type, metallic and semiconductor electron conduction, contained fluid, degree of fluid saturation, porosity and permeability, connectivity of pores, chemical inhomogeneities, depositional or crystallization conditions, temperature, and orientation of their components, particularly when insulating grains obstruct electrical paths^2^. The electrical resistivity of rocks is highly variable and can range as much as 10 orders of magnitude with variations of frequency, salinity and saturation^3–5^.

The grain orientation and grain boundary can cause the space between grains to be narrow^6^. Although the boundary between grains structure has an important effect on a material’s electrical properties, its relationship to resistivity is still unclear. The boundaries between grains in metals can enhance resistivity when grain size in relation to other scatters’ free paths becomes considerable. The shape and orientation of grains in rocks can significantly affect their electrical behavior; with grain boundaries and grain orientation all playing important roles in determining the material’s properties.

As granule length increases, the material’s electrical conductivity often decreases. This is because the mixture may have a grain surface that acts as a barrier to electron mobility, scattering electrons and lowering total conductivity. The number of grain surfaces per unit size increases as average grain length falls, increasing the possibility that electrons would disperse along different pathways and decreasing the electrical conductivity of materials with smaller particle sizes^7^.

The mixture of a semi-conductor and a semi-insulator can sometimes exhibit abnormal high values, for the dielectric constant and unusual conductivity behavior, at critical concentrations. The mixture laws (e. g., Maxwell–Wagner) that characterize the connection between the electrical conductivity of the mixture components and their effective dielectric constant help explain this abnormal behavior^8,9^. The real dielectric constant part ε′ represents the material’s ability to store electric energy, while the imaginary dielectric constant part ε′′ accounts for energy dissipation as heat due to dielectric losses. The effective permittivity of mixtures is not a static property but rather a dynamic function that varies with frequency.

The dielectric properties of the mixture can be significantly influenced by the presence of the conductor and insulator components. The actual dielectric value of the mixture can be higher or lower than the mean of the individual dielectric constants, depending on the characteristics of the components. The conductivity behavior of the mixture can also be affected by the presence of the conductor and insulator components. The effective electrical conductivity of the mixture cannot be higher than the value of the most conductive elements of the mixture. The characteristics of each component and the manner in which they interact influence the mixture’s actual electrical conductivity and dielectric value^10^.

Studies have been conducted on the impact of grain shapes on the electrical characteristics of rocks, especially in combinations containing a substantially insulating phase (insulator) and a significantly conducting phase (conductor). Rocks’ electrical and dielectric characteristics can be influenced by their grain shapes, and the impact of anisotropy on the shape of the grains on these characteristics has attracted a lot of interest. The grain boundaries can reduce a material’s electrical conductivity. In conclusion, the anisotropy in a grain’s structure, in particular, can have a big impact on a rock’s electrical properties, especially when mixing an insulating or semi- insulating phase with a conducting or semi- conducting phase.

The details in the next paragraphs will be as follows: The first will include a paragraph on Effective Medium Theory (EMT) as well as limitations on the Bruggeman and Maxwell–Wagner mixing formulas. The subsequent paragraph will discuss the advantages and disadvantages of effective medium theories specifics. The third paragraph examines at percolation theories in heterogeneous systems and compares (EMT) with Renormalization Group theory (RG). The third paragraph will go through the (EMT) system’s modeling details and illustrate how to calculate the grain sizes of prolates or needles in the model.

This study is the first to explore the theoretical direction of elongation of conductor or insulator grains in a mixture, whether parallel or perpendicular to the current stream. The impact of concentration and grain shape was the main objective of this study. We can cover electrical experimental range distinctions by modifying EMT and grain shape. Understanding how grain shape elongations impact electrical behavior can provide understanding of practical aspects of mixture electrical characteristics especially dealing with anomalous behavior.

## Effective medium theory (EMT)

The EMT provides a novel solution for the electrical characteristics of composite materials at ranged percolation thresholds. This theory gives an approximation for describing the properties of randomized mixed substances (mixtures). It has been updated to describe a broader class of composite materials with varying percolation thresholds and to study multilayer meta-materials and anisotropic meta-materials, providing a bridge to link theoretical predictions with practical realizations. Additionally, an iterative EMT has been developed for multi-component materials, showing good performance in describing composite materials.

The critical concentration in percolation theory describes the point at which there is a sharp increase in conductivity and abnormal high dielectric constant as conducting grains form continuous paths. This phenomenon is associated with interfacial polarization, which involves charge separation, migration, and relaxation around interfaces. The dielectric properties of composites near the percolation threshold exhibit non-monotonic polarization behavior, with conductivity increasing as the conductor concentration increases and the conductor cluster size increases. This behavior is attributed to interfacial polarization and the establishment of a percolated filler structure. The search results provide insights into the interplay between percolation theory, interfacial polarization, and dielectric properties in various composite materials, offering a comprehensive understanding of the critical concentration phenomenon and its implications for conductivity and dielectric constant.

The macroscopic characteristics of composite materials, such as the conductivity and the dielectric constant, can be represented using effective medium theories (EMTs). These concepts revolve around the characteristics and relative amounts of the constituent elements of the rock. Since it is practically hard to precisely calculate the values of the multiple constituents that make up a composite material, EMTs are formed by averaging the values of the constituents. The EMT provides a starting point to construct reliable electrical simulations of rocks by taking into account the material’s microstructure. It makes it possible to simulate the correlations between geometric parameters and the physical characteristics of various types of rocks, including shape, size, and conductivity parameters. The effective conductivity for various realistic rock formations found in mineralization zones and/or petroleum reservoirs can be developed with this theory, which offers a more accurate representation of complicated rock formations than traditional models. In conclusion, natural rocks’ electrical characteristics are significantly affected by the orientation and shape of their components. Grain shape modification allows variations in conductivity and dielectric constant within effective medium theories, which are based on the relative fractions and attributes of the components of the rock and successfully correlate experimental results.

When considering account changes in the grain shape of the materials, the EMT agrees with the experimental findings. This theory can predict electrical characteristics, particularly conductivity and the dielectric constant, at various concentrations and particle sizes. Here are some essential factors to consider: Conductivity rises when grain size falls, owing to the creation of conducting clusters between grains. The dielectric constant rises as the porosity or volume of air between grains reduces. The EMT provides an invaluable framework for understanding how grain shape differences affect electrical characteristics in materials. This theory, which takes consideration of grain size, grain boundaries, and other variables, may accurately predict the dielectric constant and conductivity of materials under different conditions.

The grain shape of the components has been found to have a considerable impact on the electrical characteristics of the investigated samples. The latter consequence could exceed the effect of conductor concentration itself. This effect is very essential in electrical structures (conductivity, resistivity, and dielectric constant). When AC electric field (or voltage) is applied to a material, the electrons and molecules migrate between grains and clusters, and their concentration and velocity determines its electrical conductivity. The electrical characteristics of materials are essential because they describe electrical energy conduction (and dissipation) and storage.

The effect of conductor concentration in a medium on the electrical properties of the mixture has been studied in a wide range of structures including emulsions of synthetic liquid mineral oil–water, natural rocks of Nubian sandstone-conductor, and synthetic mixtures of sand-aluminum and sand-graphite^3–5^. The dielectric properties of water-in-oil emulsions are different from the pure aqueous and pure oil constituents^11,12^. The electrical conductivity of oil-in-water emulsions has been tracked using electrical impedance.

There is little agreement among mixture laws and the results of experiments for emulsions (below the percolation threshold and at relatively low conductor concentrations), which may indicate that a mixture’s dielectric constant is mostly determined by the shapes of the conducting fragments suspended in the dielectric medium^13,14^. Mixture theories can be used for low concentrations as they take into account the fact that the host material remains host even at very high inclusion concentrations. However, mixture formulas do not anticipate excessively high dielectric constants or percolation thresholds^13^.

Bruggeman’s mixture formula^14,15^ is used to analyze the electrical properties of various systems by considering the effect of grain shape. However, traditional mixture laws (e. g. Maxwell- Wagner, and Bruggeman) are not sufficient to explain the electrical properties of natural samples that contain multiple constituents^14,15^. Furthermore, these mixture laws do not account for the effects of coatings between grains or grain size and shape on electrical behavior. Percolation theory addresses these limitations by focusing on the continuous pathways of conductive materials and the peak in the dielectric constant. For a conductive-insulator mixture, percolation theory predicts that at the critical concentration, the conductivity changes abruptly, at a certain (percolation) concentration, as the concentration of the conducting phase increases^16,17^.

The Bruggeman mixing formula and Maxwell–Wagner theory (as standard formulas) fail to account for the electrical behavior of natural samples, the influence of grain coating, grain size, or shape. Percolation theory focuses on conductive material’s continuous pathways and dielectric constant peaks. Below the percolation threshold, the system is constructed up of detached clusters, but after the threshold, an enormous connected cluster arises, allowing the compound or mixture to percolate through the system.

In a study, for binary mixtures, on natural hematitic sandstone mixtures, it was discovered that the traditional EMT, did not predict experimental results for natural rocks ^18–20^. Nevertheless, for natural rocks, the EMTs ignore the impact of more than two elements, the influence of coating between grains, and the size or shapes of the grains. EMTs, in summary, are helpful for researching the electrical characteristics of heterogeneous systems, but they have certain disadvantages when working with more than two components, different grain sizes, or different shapes.

## Theories of percolation in heterogeneous systems

Percolation theory examines how a substance flows through a porous material or how a signal propagates through a network. It is used to study a wide range of phenomena, including the flow of liquids through porous rocks, and the conductivity of random materials. The percolation theory is used to describe the behavior of conductive fillers in a conductor–insulator system. When continuous channels of the conductor start to emerge, the sample exhibits conductive behavior^1,17^. Percolation theories can predict or describe the formation of a continuous channel of inclusion at intermediate concentrations. The critical concentration is around 30% for three-dimensional systems (spherical grains) and around 50% for two-dimensional systems. Percolation theory defines the interconnection of conductive fillers within a matrix. Above the percolation threshold, conductivity is high, whereas below it, conductivity is (no conduction connections) zero^21^. Near the percolation threshold, the dielectric constant of a system with alternating current becomes extremely high because the difference in distance between the conductor grains is very small, and the system acts like gigantic capacitors^8,9,22^.

When the percolation threshold is achieved, the material creates a more dense structure, resulting in higher charge accumulation and an increasing dielectric constant. The percolation threshold is the minimum volume fraction of a conductor that is needed to form a continuous conducting path within a given material. The percolation threshold models help predict the electrical properties values near the percolation threshold, which can be used to optimize the performance of AC electrical properties in materials^23–25^.

The EMT is an approach to characterize the properties of composite substances using the characteristics and relative percentages of their component parts. In this theory, a heterogeneous system is represented by a uniform electrical network that reflects the mixture’s unknown effective features^26,27^. Local electric elements representing the mixed phases are injected into the network, resulting in local distortions. The equation of the effective medium is established by assuming that the average of these local distortions resulting from the system phases with their corresponding concentrations is zero^17,28,29^.

Renormalization group (RG) theory is an effective tool for analyzing hierarchical scales and symmetries in dynamic structures including heterogeneous networks. The fundamental concept of RG is to examine how the physical behavior of a system varies as its scale is altered in relation to criticality. When dealing with heterogeneous networks, the renormalization process involves generating the structure of the network at small sizes, substituting a single node for every connected node block, and gradually creating larger grains until the entire system can be generated in a few steps.

EMT and renormalization group method try to solve the percolation problem. Each of these theories has advantage and disadvantages^26,27^. EMT advantages is that it can take into account the effect of grain shape, which is important in some percolation problems; and useful for determining percolation thresholds in random heterogeneous materials. The disadvantages are that it may not be suitable for all percolation problems, as it focuses on the effect of grain shape. The Renormalization Group Theory advantages is that it can handle the effect of grain size, which is important in some percolation problems; and can be applied to time-varying networks. The disadvantage is that it may not be suitable for all percolation problems, as it focuses on the effect of grain size^30,31^.

In a previous article^23,30,31^ we tried to show the impact of some limited grain shape elongation perpendicular to the direction of the current (sphere, prolate spheroid, ellipsoidal, disk, and needle). We will now attempt to demonstrate the extended effect of the elongation of the grains (sphere, oblate spheroid, ellipsoidal, disk, and needle) in the direction of the current and perpendicular to its direction.

In conclusion, EMT is more appropriate for analyzing the effect of grain shape, whereas renormalization group theory is better suited for considering the effect of grain size.

## Modeling of the system

Prolates or needles are designated as elongated in the direction of a line joining the poles or the long axis of it is presented at the direction of the current. Oblates or discs are designated as flattened or depressed at the poles or the long axis of it is presented perpendicular to the direction of the current (Table 1). When prolate or oblate spheroids are subjected to the influence of an alternating electric field, their electrical characteristics differ tremendously.

**Table 1:** Shows the conducting grains extend with the stream of current and the rough critical percolation concentration values.

Conducting grain shapes between electrodes	Percolation concentration
	Very small
	Small
	Small
	Moderate
	Higher
	Very high
	Extremely high

The effective complex dielectric constant is given by^22^,

1 $$\varepsilon^{*} = \varepsilon{\prime} + \frac{\sigma }{{iw\varepsilon_{0} }}$$

The EMT for spherical grains impeded in a medium of spherical grains provides an expression for the effective complex dielectric constant as

2 $$\sum\limits_{\alpha } {f_{\alpha } } \frac{{\varepsilon^{*} - \varepsilon_{\alpha }^{*} }}{{2\varepsilon^{*} + \varepsilon_{\alpha }^{*} }} = 0$$

where $(\varepsilon_{0} )$ is the permittivity of vacuum, real dielectric constant $\varepsilon{\prime}$, $(\sigma )$ is the conductivity $(w)$ is the angular frequency, complex dielectric constants of the constituents is $(\varepsilon_{\alpha }^{*} )$ and $(f_{\alpha } )$ is the volume fraction of each constituent $(\alpha )$.

In the case when the grains have spheroidal shapes, Eq. (2), can be written in the form ^22^,

3 $$\frac{1}{3}\sum\limits_{\alpha } {\sum\limits_{i = 1}^{3} {\left\langle {\frac{{\varepsilon_{\alpha }^{*} - \varepsilon^{*} }}{{L_{\alpha i} \varepsilon_{\alpha }^{*} + (1 - L_{\alpha i} )\varepsilon^{*} }}} \right\rangle } } f_{\alpha } = 0$$

This formula takes into consideration the random orientations of the grains in the x, y, and z directions $\left( {i = { 1, 2, 3}} \right)$ and $L_{\alpha i}$ is the depolarization factor of $\alpha^{th}$ constituent in the $i^{th}$ direction.

**Fig. 1:**
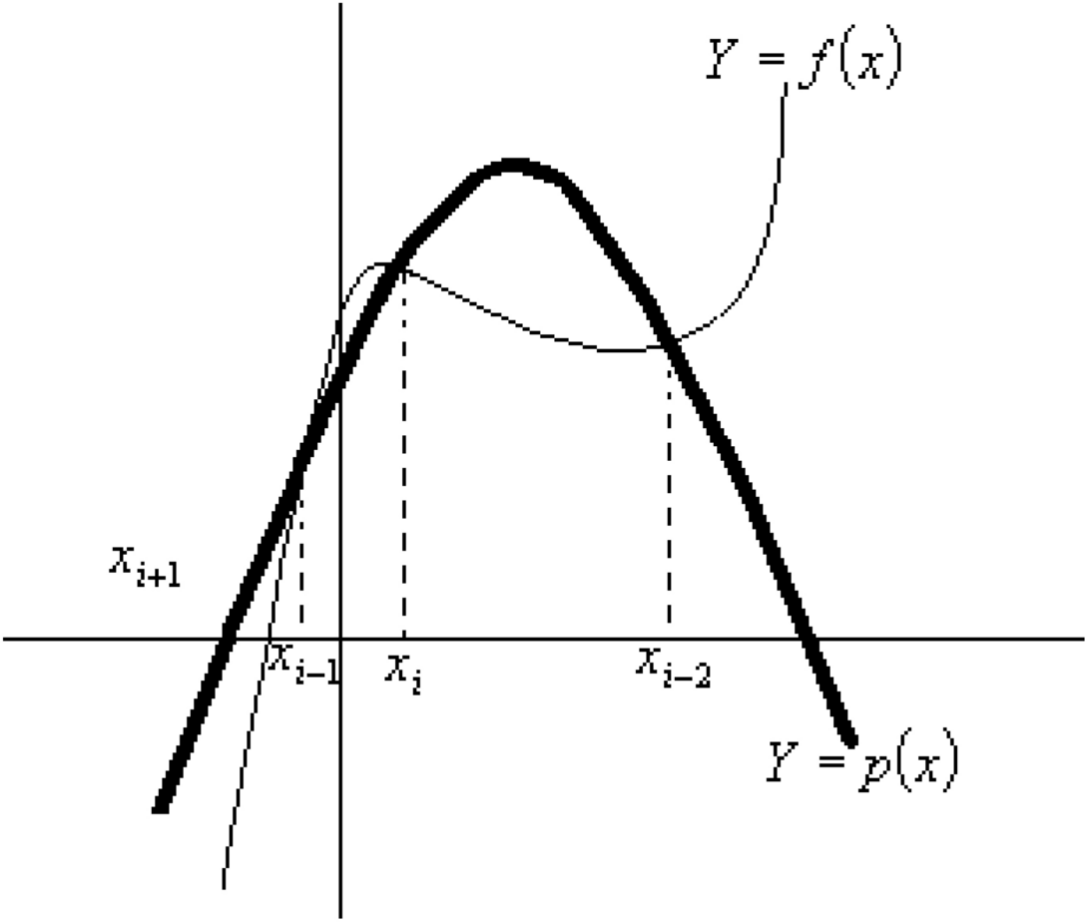
Müller’s method.

In order to solve the previous nonlinear equation (Eq. 3) to obtain $\varepsilon^{*}$, Müller’s method, Fig. 1, is used^32^. This method is similar to the secant method in which we determine, from the approximations *x*_*i*_, *x*_*i-*1_ for a root of *f*(*x*) = 0, the next approximation *x*_*i+*1_ as the zero of the linear polynomial *p*(*x*) which goes through the two points {*x*_*i*_, *f*(*x*_*i*_)} and {*x*_*i-*1_, *f*(*x*_*i-*1_)}. In Müller’s method, the next approximation, *x*_*i+*1_, is found as a zero of the parabola defined by *p*(*x*), which goes through the three points {*x*_*i*_, *f*(*x*_*i*_)}, {*x*_*i-*1_, *f*(*x*_*i-*1_)} and {*x*_*i-*2_, *f*(*x*_*i-*2_)}. The three points are used as approximations for a root of *f*(*x*), and a zero of the equation of the parabola *p*(*x*) gives an improved solution, Fig. 4. The process is then repeated using *x*_*i-*1_, *x*_*i*_, *x*_*i+*1_ as the three basic approximations.

The values of $L_{\alpha i}$ are determined by the eccentricity e of the spheroid. For prolate spheroids (~ needles), with semiaxes ${\text{b}}_{{1}} > {\text{b}}_{{2}} = {\text{b}}_{{3}}$ ^33^, $e = \left[ {1 - \mathop {\left( {\frac{{b_{2} }}{{b_{1} }}} \right)}\nolimits^{2} } \right]^{1/2}$, and the depolarization factor in the direction of the spheroid axis of symmetry is

4 $$L_{1} = \frac{{1 - e^{2} }}{{2e^{3} }}\left( {\ln \frac{1 + e}{{1 - e}} - 2e} \right)$$

For oblate spheroids (~ discs), with ${\text{b}}_{{1}} < {\text{b}}_{{2}} = {\text{b}}_{{3}}$, $e = \left[ {\mathop {\left( {\frac{{b_{2} }}{{b_{1} }}} \right)}\nolimits^{2} - 1} \right]^{1/2}$ and

5 $$L_{1} = \frac{{1 + e^{2} }}{{e^{3} }}\left( {e - \tan^{ - 1} e} \right)$$

With $$L_{1} + L_{2} + L_{3} = 1$$,$$L_{2} = L_{3}$$

For spherical grains $$L_{1} = L_{2} = L_{3} = {\raise0.5ex\hbox{$\scriptstyle 1$} \kern-0.1em/\kern-0.15em \lower0.25ex\hbox{$\scriptstyle 3$}}$$.

For needles $$L_{1} \to 1,L_{2} = L_{3} \approx 0.$$

For discs $$L_{1} \to 0,L_{2} = L_{3} \approx \frac{1}{2}$$.

The method does not require an exact initial solution^32^, is iterative, does not require evaluation of the derivatives of the function, and preserves real and complex roots. To obtain an initial solution to the correct roots (in each of the roots of the equation), one needs to start with a unit volume of an insulator (or semiconductor) for which the exact solution is known at each frequency Fig. 1. Increase the volume fraction of the semiconductor by a small value and use the solution of the previous concentration as the starting solution. It is assumed that the increase in concentration near the percolation threshold is very small because the changes in dielectric constant and conductivity occur very quickly. Above the percolation threshold, larger increments are used because the changes in dielectric constant and conductivity are small. The method is iterated to obtain the dielectric constant for each concentration value ^34^.

## Results and discussion

The mixture laws are typically capable of predicting the experimental results of emulsions. However, they typically lack the ability to predict the experimental results for solid mixtures of conductor and insulation except at low concentrations of the latter (impurities). The theory of percolation can account for the majority of the electrical properties of solids and random collections ^34^. The effect of the grain’s shape and the random grain orientations are taken into consideration when using EMT.

The EMT will be utilized in this context to predict the electrical properties of substances. The samples are considered a mixture of semiconductor and semi-insulator that is heterogeneous. The elements’ shape of grain may have a greater impact on the conductor than the concentration of water or salinity. It will be tried to deal with potential variations of the measured A. C. dielectric constant and conductivity at different concentrations, in the direction of lengthening, of conductor, or insulator grains in the direction or perpendicular to the current direction, within the frame of grain shape variations using EMT. In a previous article ^23^, we tried to show the impact of some limited grain shape elongation perpendicular to the direction of the current. Now, we will try to show the extended effect of the grain shape elongation of the grains in the direction of the current and perpendicular to the direction of it ^35–37^.

**Fig. 2:**
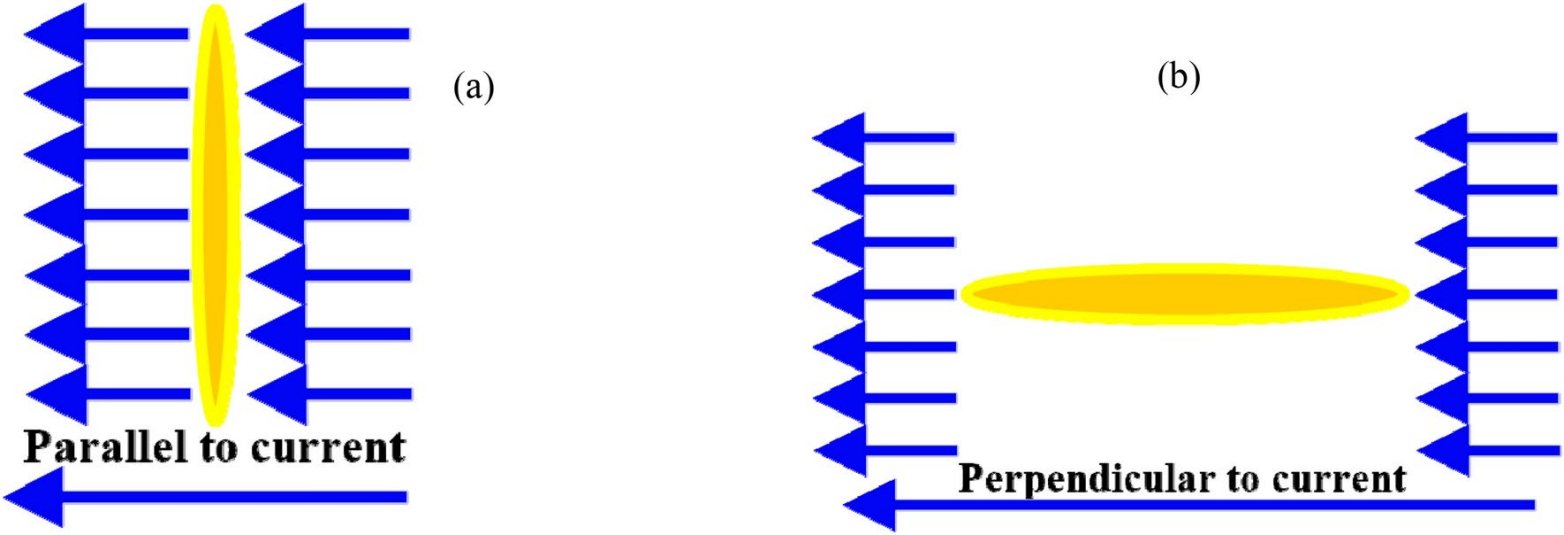
Depicts the elongation of the grains’ long axis in two directions: (**a**) parallel to the current stream and (**b**) perpendicular to it.

**Fig. 3:**
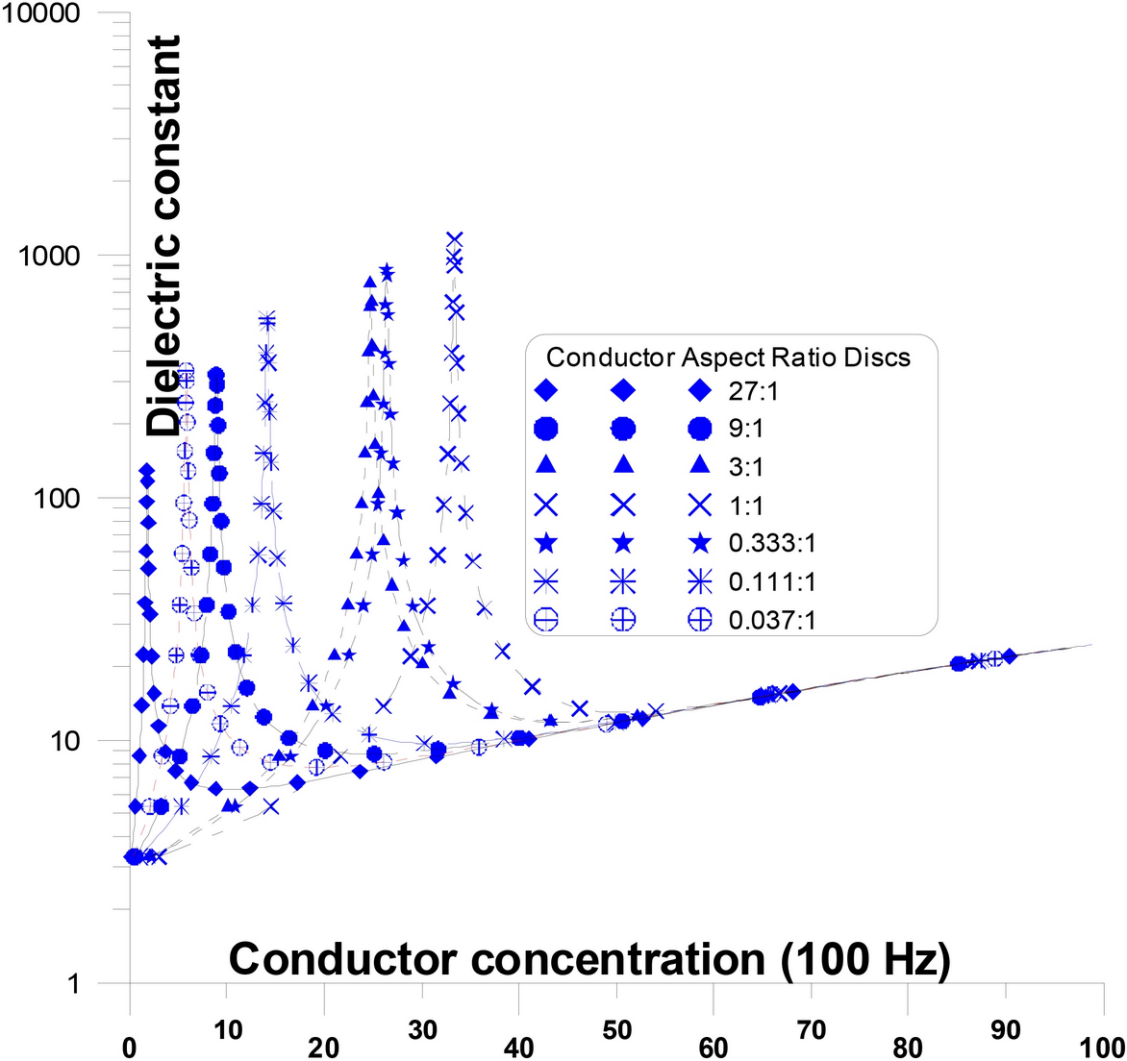
Dielectric constant as a function of various conductor concentration and conductor aspect ratio discs in parallel to the stream of the current at a frequency of 100 Hz.

Figure 2 depicts the elongation of the grains’ long axis in two directions: (a) parallel to the current stream and (b) perpendicular to it. When we address about axis elongation, whether parallel or perpendicular to the current stream, we mean the long axis with respect to the other two tiny axes. The granular structure of the ingredients (Figs. 2 and 3) show a comparison of the dielectric constant and conductivity of mixtures with varying conductor concentrations at a frequency of 100 Hz ^38^. Figure 2 shows the dielectric constant as a function of various conductor concentration and conductor aspect ratio discs in parallel to the stream of the current at a frequency of 100 Hz. The influence of component concentration and granular shape is demonstrated using the EMT for randomly oriented spheroidal grains at 100 Hz. Spheroidal conductor grains with varying aspect ratios are employed in other insulator phase ^1,21,39,40^.

Figure 3 shows that modification in the shapes (aspect ratios) of conductor grains in parallel to the stream of the current has a significant impact on the enhancement of the mixture dielectric constant and the concentration at which the enhancement occurs (critical concentration peak). Equations 3, 4, and 5 are used to plot these curves (for dielectric constant and conductivity). The enlargement of spherical conductor grains dielectric constant values, occur at a critical conductor concentration of around 33% ^41^.

Conductor discs Fig. 2 with varying aspect ratios (from 27:1 to 0.037:1) are present with the (parallel) stream of current. At the figure Fig. 2 where the aspect ratio is changed, the dielectric constant’s general response with concentration is replicated. The dielectric constant rises slowly initially (below $P_{C}$) with an increase in conductor concentration and abnormally at relatively higher conductor concentrations until it goes above the percolation threshold ($P_{C}$). The dielectric constant starts to drop sharply beyond the percolation threshold (above $P_{C}$) and ultimately approaches the conductor’s dielectric constant value ^37,38^. The conductor’s dielectric constant value is nearly (25), Fig. 3. This may be the value of wet clay or a shale or Water-Saturated Mudstones (Keller and Licastro 1957, Garrouch et al. 2009, Nigel and Cassidy 2009). The percolation threshold originates at lower conductor concentrations (~ 3%) when the aspect ratio of the conductor is relatively high (27:1), thereby improving the possibility of early continuous conducting pathways occurring between electrodes (Table 2). The percolation threshold originates at greater conductor concentrations (shifts from ~ 5% to ~ 33%) when the aspect ratio of the conductor falls (from 27:1 to 1:1). For this reason, with greater aspect ratio of the conductor concentrations, the possibility of early continuous conducting pathways between electrodes is improved. The values of the dielectric constant peak ($P_{PC}$), at 100 Hz, range from 150 to 1050. The percolation threshold for the other conductor concentrations decreases again and shifts from ~ 28% to ~ 7% when the aspect ratio of the conductor falls from 0.333:1 to 0.037:1. For this reason, with greater aspect ratio of the conductor concentrations, the possibility of late continuous conducting pathways between electrodes are improved ^42^. The values of the dielectric constant peak ($P_{PC}$), at 100 Hz, drop from 950 to 360. This is the reverse of the preceding example, characterized by the conductor’s long axis shifting from perpendicular to parallel to the streaming current ^43–46^.

**Table 2:** Shows the aspect ratios of the grains, at each figure, at a certain frequency, the critical threshold and the Dielectric constant peak.

Aspect ratio	Critical threshold	Case	Dielectric constant peak	Frequency	Figure
27:1	1.8	Conductor in insulator at the same direction of the current	108.96	100 Hz	Figure 3
9:1	8.9		324.92		
3:1	24.74		763.81		
1:1	33.33		1156.7		
0.333:1	26.403		898.96		
0.111:1	14.12		567.04		
0.037:1	5.7967		337.79		
–	–		No peak	10^7^ Hz	Figure 4
9:1	35.403	Insulator in insulator at the same direction of the current	1254.4	100 Hz	Figure 5
3:1	34.458		1209.9		
1:1	33.33		1158.8		
0.333:1	36.558		1342.7		
0.111:1	49.201		2150.9		
0.037:1	69.026		3804.8		
0.012:1	85.778		6675.2		
0.004:1	94.604		11,641		
0.001:1	98.105		20,103		
0.0005:1	99.359		34,584		
–	–		No peak	10^7^ Hz	Figure 6
27:1	1.8	Conductor in insulator at the same direction of the current		100 Hz	Figure 7
9:1	8.9				
3:1	24.74				
1:1	33.33				
0.333:1	26.403				
0.111:1	14.12				
0.037:1	5.7967				
–	–		No peak	10^7^ Hz	Figure 8
9:1	35.403	Insulator in insulator at the same direction of the current	1254.4	100 Hz	Figure 9
3:1	34.458		1209.9		
1:1	33.33		1158.8		
0.333:1	36.558		1342.7		
0.111:1	49.201		2150.9		
0.037:1	69.026		3804.8		
0.012:1	85.778		6675.2		
0.004:1	94.604		11,641		
0.001:1	98.105		20,103		
0.0005:1	99.359		34,584		
–	–		No peak	10^7^ Hz	Figure 10

**Fig. 4:**
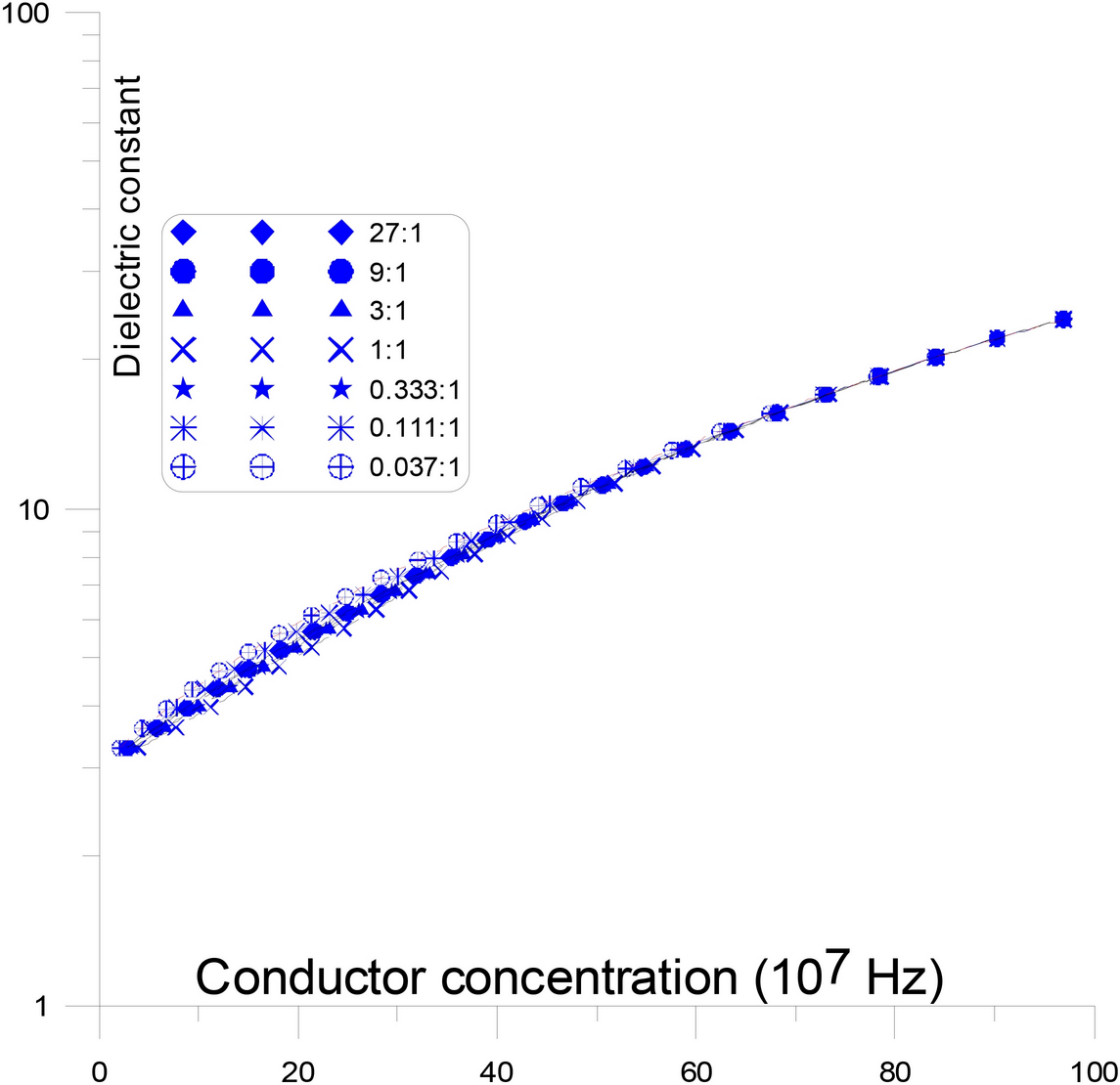
Dielectric constant as a function of different conductor concentration, with different conductor aspect ratio discs, perpendicular to the stream of current, at frequency (10^7^ Hz).

The existence of insulator discs (perpendicular to the stream of the current) prevents the conductor material from building continuous pathways at lower conductor concentrations. As the insulator discs (perpendicular to the current) become thinner, thin insulating clusters (or equivalently coating clusters) develop between the conductor grains, developing the dielectric constant and increasing percolation concentration ^34^. On the other hand, substituting conductor discs with needles (perpendicular to the current) helps minimize the space between conductor continuous pathways, resulting in an increase in the dielectric constant that may be comparable to insulator discs.

The presence of insulator discs (in parallel to the stream of the current) does not affect the conductor continuous paths at any conductor concentrations, because it (insulator) does not cut any continuous conductor clusters ^47,48^. As the insulator discs (in parallel to the stream of the current) become thinner or thicker they constitute insulating cluster lines (thin or thick) between the conductor grains (or between the electrodes) which does not make any considerable changes at the dielectric constant and accordingly, does not affect the percolation concentration values. On the other hand, the existence of conducting discs or needles (in the current’s direction) causes continuous conductor pathways to develop between the electrodes. Moving away from conductor discs to needles allows for the formation of conductor continuous pathways at lower conductor concentrations, which causes an increase in the dielectric constant but at a smaller magnitude than with insulator discs ^49^.

Figure 4 displays the fluctuations of the dielectric constant as a function of changing conductor concentration, with discs representing the different conductor aspect ratios, in parallel to the stream of current, at frequency (10^7^ Hz). At this figure, conductor discs with varying aspect ratios (from 9:1 to 0.037:1) are present in parallel to the stream of current. When the aspect ratio is changed, the dielectric constant’s overall response to concentration is nearly replicated (almost all the curves are same). The dielectric constant gradually rises with an increase in conductor concentration from the value of the insulator dielectric constant (10^7^ Hz) until it reaches the other conductor dielectric constant value at the same frequency, with no percolation threshold peak ^50–53^. This is typical as high frequencies enable low-energy electrons to pass through energy barriers, which is why the dielectric constant does not exhibit peaks at these frequencies.

The conductor material acts like a semi-conductor as a result. It is noticed that, at low conductor concentrations, as the aspect ratios of the conductor increase (from 9:1 to 0.037:1) the dielectric constant value increases slowly and smoothly. This is argued due to the fact that the elongation of the conductor aspect ratios, in parallel to the stream of current, at frequency (10^7^ Hz), are recorded as semi-conductor and the aspect ratios does not make much changes to the current. This causes in parallel material to behave like a semi-conductor. It is observed that the dielectric constant value grows gradually and smoothly at low conductor concentrations as the conductor’s aspect ratios changes (from 9:1 to 0.037:1). This is disputed since the disputed aspect ratios at a frequency of 10^7^ Hz, which are elongated in parallel to the current stream, are recorded as semi-conductors and do not significantly, alter the current ^54–56^.

**Fig. 5:**
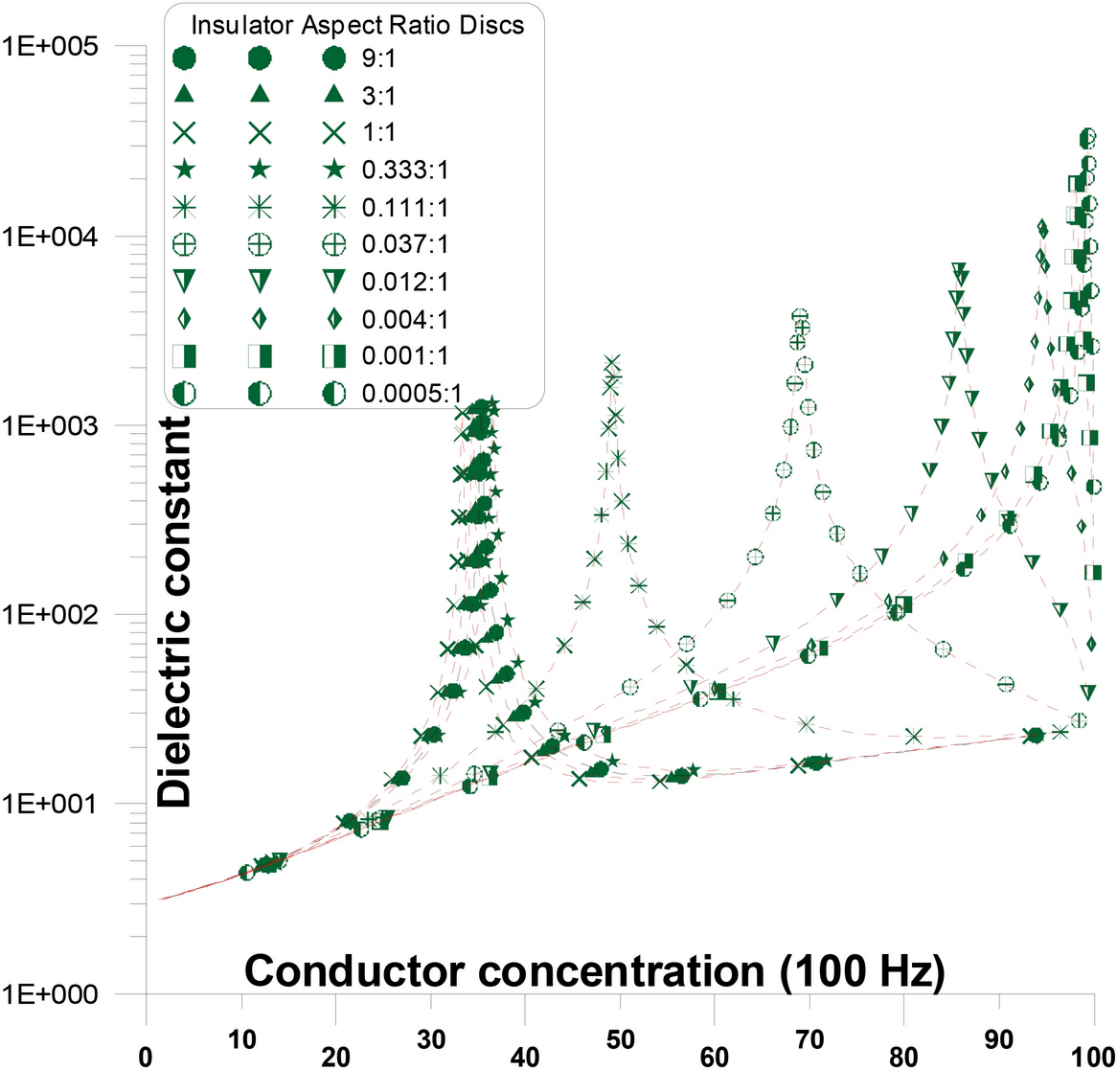
Dielectric constant as a function of various conductor concentration and insulator aspect ratio discs in parallel to the stream of the current at a frequency of 100 Hz.

Figure 5 demonstrates the influence of grain shape on the dielectric constant of an insulator-semiconductor combination as well as the extremely high dielectric constant values achieved experimentally using EMT at 100 Hz. For one phase (insulator or semiconductor), spheroidal grains with varying aspect ratios are used, while spherical grains are employed for the other phase. Figure 5 demonstrates that the degree of enhancement of the mixture dielectric constant and the concentration at which the enhancement occurs are significantly influenced by the variation in the shape of the grains. The increase occurs at a critical concentration of about 33% for spherical grains. At intermediate semi-conductor concentrations, the semi-conductor material’s capability to form continuous channels is obstructed by the presence of insulator discs. The thinner the insulator discs are, the thinner the insulating layers that develop between the grains of the semi-conductor, enhancing the dielectric constant and enhancing the percolation concentration. However, in contrary to insulator discs or spheres, the presence of semiconductor needles or discs enables semiconductor continuous pathways to develop at low semi-conductor concentrations, which again enhances the dielectric constant but at lower values ^57–59^.

Because the insulator discs obstruct the semiconductor’s pathways, the critical concentration grows as we proceed from insulator spheres (critical concentration ~ 33%) to insulator discs. Since they won’t obstruct the conductor routes, insulator needles won’t be of the utmost significance. Transitioning from semi-conductor spheres (critical concentration approximately 33%) to semi-conductor needles or discs implies an overall reduction in critical concentration. This is because semi-conductor needles generate an early percolation threshold, whereas semiconductor discs accomplish the same effect at slower rates ^3–5^.

The impact of particle size and shape on the dielectric characteristics of materials can be responsible for the extremely high dielectric constant values measured experimentally at 100 Hz, which are not predictable from mixture laws. Studies have indicated that the dielectric constant of substances is significantly influenced by the size of their grains. Furthermore, the dielectric characteristics of materials are significantly influenced by the crystalline structure, microstructure, and grain size.

From Fig. 5, it is clear that the concentration at which enhancement takes place and the improvement of the mixture dielectric constant are both greatly influenced by differences in the insulator grains’ shapes. At high conductor concentrations, continuous pathways form when the insulator discs prevent conductor material from generating disconnected pathways ^26,27,60^.

The EMT evaluates how apparent transport properties of composite materials, such as rocks, and porous media, are modified by grain size, grain borders, and contrast between phases. The EMT establishes a framework for recognizing how the overall conduction properties of the composite material, including the generation of continuous pathways at high conductor concentrations, are influenced by the geometry of the grains. As a result, the EMT provides insightful information about how modifications in grain shape relate to the electrical characteristics of composite materials.

The dielectric constant is significantly increased when the insulator discs are thinner and generate a thin insulating layer (the covering layer) between the conductor grains. However, the existence of conductor needles or discs permits conductor continuous pathways to emerge at low concentrations of conductors, which again increases the dielectric constant. It was feasible to cover the experimental range of alterations of the dielectric constant at various concentrations within the frame of grain shape variations employed here. As a result, an insulator’s dielectric constant is significantly affected by particle size, shape, and the presence of conductive components, all of which impact its electrical properties ^33,61–65^.

**Fig. 6:**
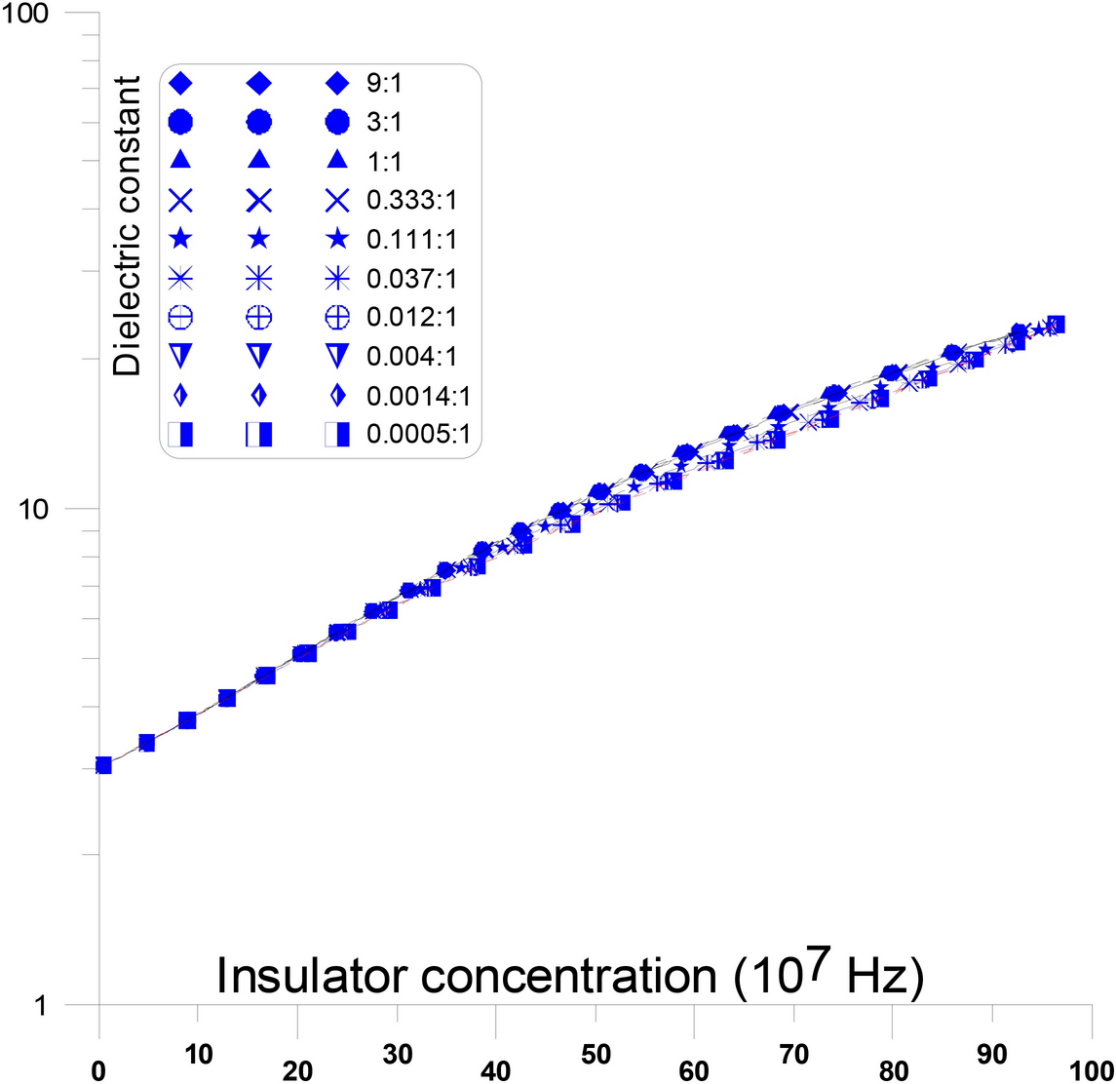
Dielectric constant as a function of different conductor concentration, with different insulator aspect ratio discs, perpendicular to the stream of current, at frequency (10^7^ Hz).

Figure 6 displays the fluctuations of the dielectric constant as a function of changing conductor concentration, with discs representing the different insulator aspect ratios, perpendicular to the stream of current, at frequency (10^7^ Hz). At this figure, dielectric constant discs with varying aspect ratios (from 9:1 to 0.0005:1) are present perpendicular to the stream of current. When the aspect ratio is changed, the dielectric constant’s overall response to concentration is nearly replicated (almost all the curves are same). The dielectric constant gradually rises with an increase in insulator concentration from the value of the insulator dielectric constant (10^7^ Hz) until it reaches the other conductor dielectric constant value at the same frequency, with no percolation threshold peak. This is typical as high frequencies enable low-energy electrons to pass through energy barriers, which is why the dielectric constant does not exhibit peaks at these frequencies ^66–69^.

The insulator material acts like a semi-conductor as a result. It is noticed that, at low insulator concentrations, as the aspect ratios of the insulator increase (from 9:1 to 0.0005:1) the dielectric constant value increases slowly and smoothly. This is argued due to the fact that the elongation of the insulator aspect ratios, perpendicular to the stream of current, at frequency (10^7^ Hz), are recorded as semi-conductor and the aspect ratios does not make much changes to the current.

This causes the insulator material to behave like a semi-insulator. It is observed that the dielectric constant value grows gradually and smoothly at low insulator concentrations as the insulator’s aspect ratios changes (from 9:1 to 0.0005:1). This is disputed since the insulator aspect ratios at a frequency of 10^7^ Hz, which are elongated perpendicular to the current stream, are recorded as semi-conductors and do not significantly, alter the current ^8,70–73^.

**Fig. 7:**
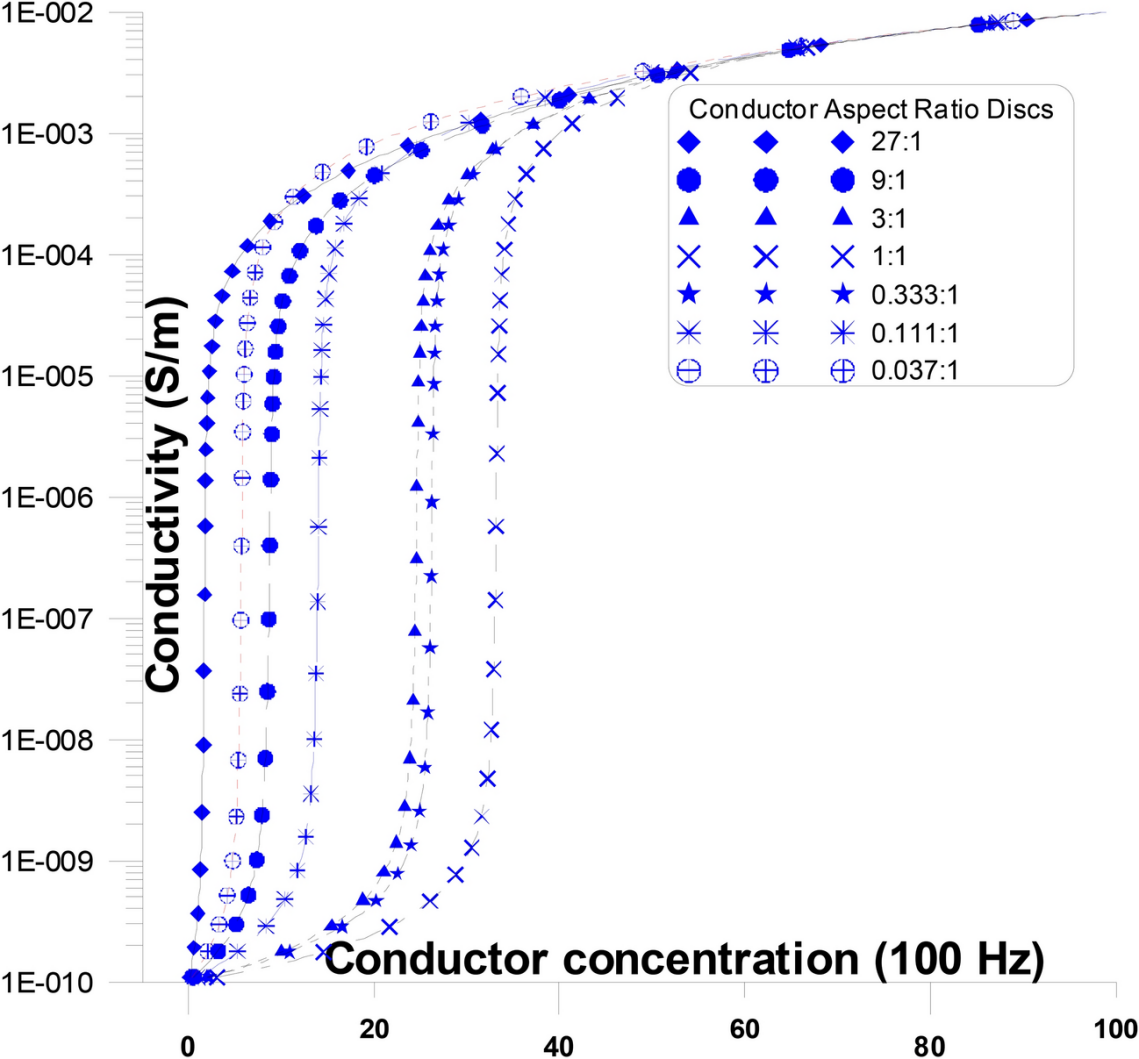
Conductivity as a function of changing conductor concentration and conductor aspect ratio discs in the current direction at a frequency of 100 Hz.

Figure 7 displays the fluctuations of the conductivity as a function of various conductor concentration and conductor aspect ratio discs in the current’s direction at a frequency of 100 Hz. At this figure, conductor discs with varying aspect ratios (from 27:1 to 0.037:1) are present with the stream of current. At the figure where the aspect ratio is changed, the conductivity’s general response with concentration is replicated. The conductivity rises (from the insulator value) slowly initially with an increase in conductor concentration and abruptly at relatively higher conductor concentrations until it goes above the percolation threshold. The conductivity starts to be stable (at the conductive value) at the conductor concentrations beyond the percolation threshold and ultimately approaches the conductivity value of the conductor of the mixture. The percolation threshold originates at lower conductor concentrations (~ 1.8%) when the aspect ratio of the conductor is relatively high (27:1), thereby indicating the possibility of early continuous conducting pathways occurring between electrodes. The percolation threshold, at greater conductor concentrations, shifts from ~ 1.8% to ~ 33.3%, when the aspect ratio of the conductor falls, from 27:1 to 1:1. For this reason, with greater aspect ratio of the conductor concentrations, the possibility of early continuous conducting pathways between electrodes is improved. The percolation threshold, at greater conductor concentrations, shifts from ~ 33.3% to ~ 5.7%, when the aspect ratio of the conductor falls, from 1:1 to 0.037:1. The enlargement of spherical conductor grains dielectric constant values, occur at a critical conductor concentration of around 33.3%.

Conductor discs Fig. 7 with varying aspect ratios (from 27:1 to 0.037:1) are present with the (parallel) stream of current. At the figure Fig. 7 where the aspect ratio is changed, the conductivity’s general response with concentration is replicated. The conductivity rises slowly initially (below $P_{C}$) with a small increase in conductor concentration and abnormally at relatively higher conductor concentrations until it goes above the percolation threshold ($P_{C}$). The conductivity starts to saturate beyond the percolation threshold (above $P_{C}$) and ultimately approaches the other conductor’s conductivity value. The percolation threshold originates at low conductor concentrations (~ 1.8%) when the aspect ratio of the conductor is relatively high (27:1), thereby improving the possibility of early continuous conducting pathways occurring between electrodes Table 2. The percolation threshold originates at greater conductor concentrations (shifts from ~ 1.8% to ~ 33.3%) when the aspect ratio of the conductor falls (from 27:1 to 1:1). For this reason, with greater aspect ratio of the conductor concentrations, the possibility of early continuous conducting pathways between electrodes is improved. The percolation threshold for the other conductor concentrations decreases again and shifts from ~ 33.3% to ~ 5.8% when the aspect ratio of the conductor falls from 1:1 to 0.037:1. For this reason, with greater aspect ratio of the conductor concentrations, the possibility of late continuous conducting pathways between electrodes are improved. The conductor’s long axes are present in the first cases (27:1 to 1:1) parallel to the streaming current then the long axes are (1:1 to 0.037:1) present perpendicular to the streaming current.

The existence of the conductor’s long axes (parallel to the streaming current) motivates the conductor material to build continuous conductor pathways at relatively lower conductor concentrations (~ 1.8% at 27:1). As the conductor’s long axes (parallel to the streaming current) become smaller, less conducting clusters may be developed between the conductor grains, then increasing percolation concentration (from ~ 1.8% at 27:1 to ~ 33.3% at 1:1).

On the other hand, as the conductor’s long axes (perpendicular to the streaming current) become smaller, less conducting clusters may be developed between the conductor grains, then decreasing percolation concentration (from ~ 33.3% at 1:1 to ~ 5.8% at 0.037:1). These conductor needles (perpendicular to the current) minimize the space between conductor continuous pathways, resulting in a decrease in the conductivity that may be comparable to the presence of conductor needles.

The presence of insulator discs (in parallel to the stream of the current) does not affect the conductor continuous paths at any conductor concentrations, because it (insulator) does not cut any continuous conductor clusters. As the insulator discs (in parallel to the stream of the current) become thinner or thicker they constitute insulating cluster lines (thin or thick) between the conductor grains (or between the electrodes) which does make considerable changes at the conductivity and accordingly, affect the percolation concentration values. On the other hand, the existence of conducting discs or needles (in the current’s direction) causes continuous conductor pathways to develop between the electrodes. Moving away from conductor discs to needles allows for the formation of conductor continuous pathways at lower conductor concentrations, which causes an increase in the conductivity but at a smaller magnitude than with insulator discs.

**Fig. 8:**
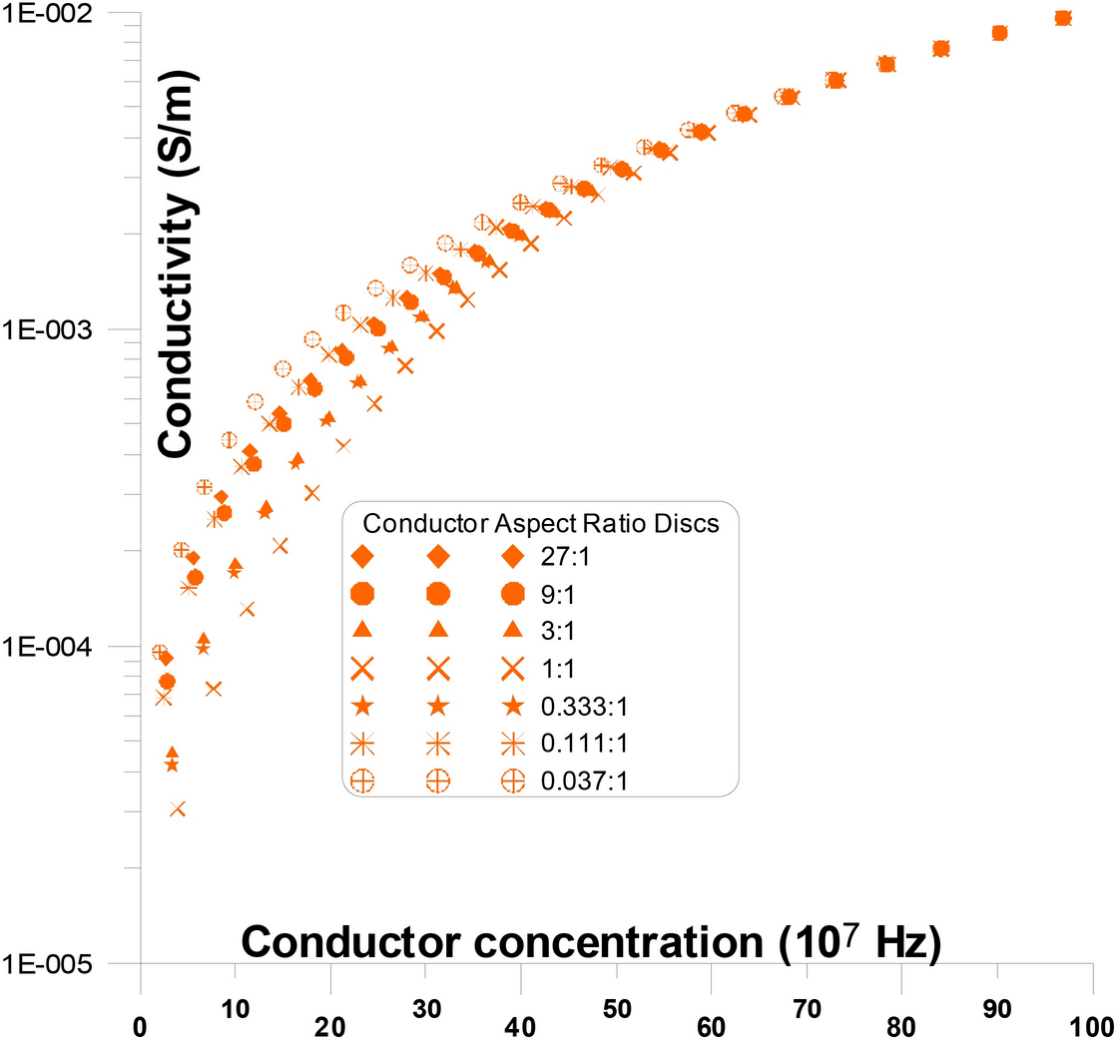
Conductivity as a function of changing conductor concentration, with discs representing the different conductor aspect ratios, with the stream of current, at frequency (10^7^ Hz).

Figure 8 displays the fluctuations of the conductivity as a function of changing conductor concentration, with discs representing the different conductor aspect ratios, with the stream of current, at frequency (10^7^ Hz). At this figure, conductor discs with varying aspect ratios (from 27:1 to 0.037:1) are present with the stream of current. When the aspect ratio is changed, the conductivity’s overall response to concentration is replicated. The conductivity gradually rises with an increase in conductor concentration from the value of the low conductor concentration conductivity (high frequency) until it reaches the high conductor concentration conductivity values, with no percolation threshold peak. It is noticed that, at low conductor concentrations, as the aspect ratios of the conductor increase (from 27:1 to 1:1, at a certain concentration) the conductivity value decreases slowly, then from (1:1 to 0.037:1) it increases slowly.

The conductor material acts like a semi-conductor as a result. It is noticed that, at low conductor concentrations, as the aspect ratios of the conductor increase (from 27:1 to 0.037:1) the conductivity value increases slowly and smoothly with the increase of conductor concentration. This is argued due to the fact that the elongation of the conductor aspect ratios, in parallel to the stream of current, at frequency (10^7^ Hz), are recorded as semi-conductor and the aspect ratios does not make much changes to the current. This causes in low conductor concentration materials to behave like a semi-conductor. It is observed that the conductivity values grow gradually and smoothly with conductor concentration at 10^7^ Hz.

**Fig. 9:**
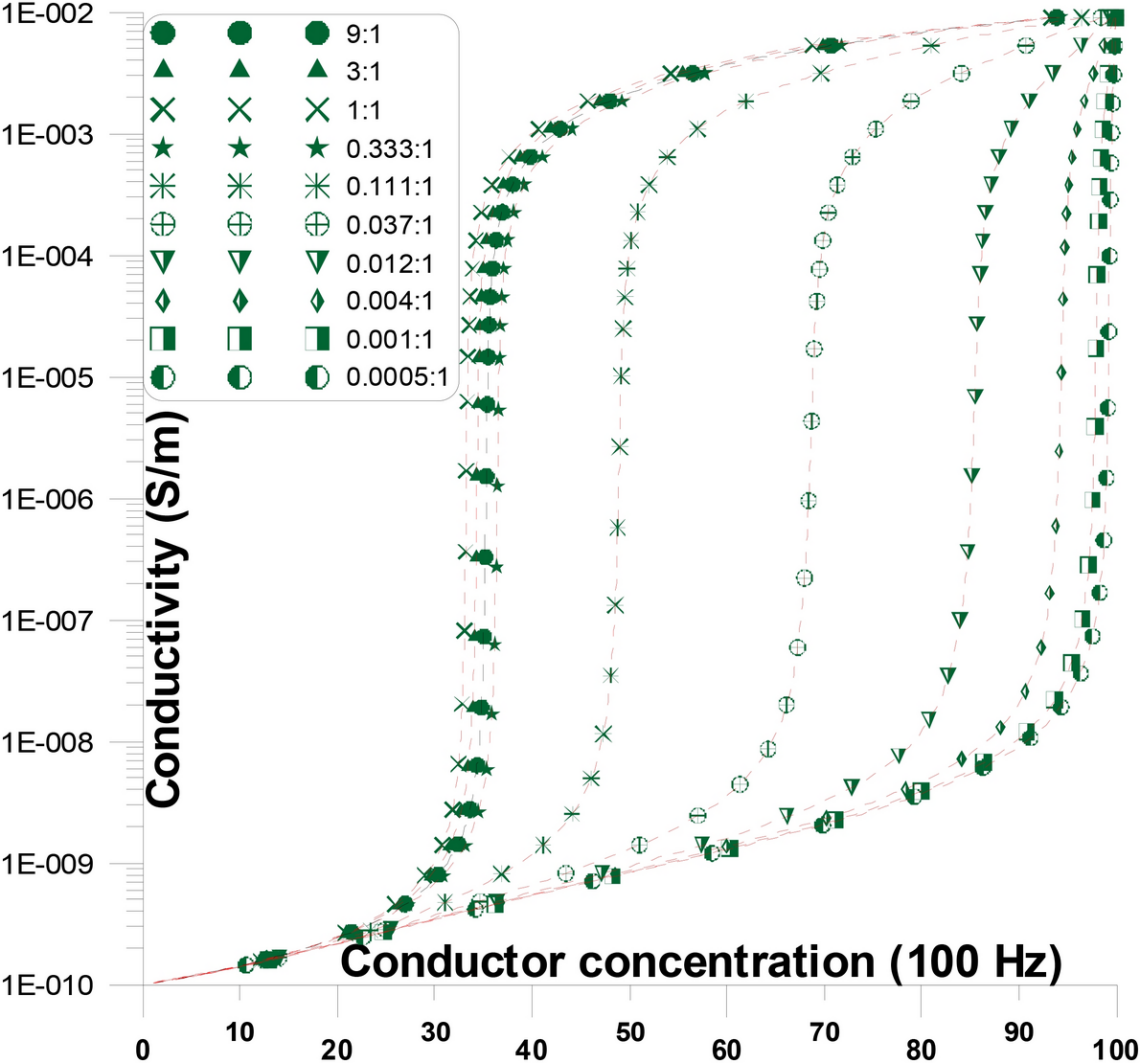
Conductivity as a function of varying conductor concentration and insulator aspect ratio discs, in parallel to the stream of current, at frequency of 100 Hz.

Figure 9 displays the fluctuations of the conductivity as a function of various conductor concentration and insulator aspect ratio discs in the current’s direction at a frequency of 100 Hz. At this figure, insulator discs with varying aspect ratios (from 9:1 to 0.0005:1) are present in parallel to the stream of current. At the figure where the aspect ratio is changed, the conductivity’s general response with concentration is always repeated. The conductivity rises slowly initially with an increase in conductor concentration and it rises abnormally at higher insulator concentrations as it goes above the percolation threshold. The conductivity starts to be stable at conductor concentrations beyond the percolation threshold and ultimately approaches the conductivity value of the conductor of the total mixture. The percolation threshold originates at lower conductor concentrations (~ 35%) when the aspect ratio of the insulator is relatively high (9:1), thereby leading to the possibility of early continuous conducting pathways occurring between electrodes. The percolation threshold, at greater aspect ratio of the insulator concentrations, shifts from ~ 33.3% at 1:1 aspect ratio. When the aspect ratio of the insulator falls from 1:1 to 0.0005:1 the critical concentration shifts from ~ 33.3% to 99.4%. For this reason, with greater aspect ratio of the insulator concentrations, the possibility of early continuous conducting pathways between electrodes is retarded.

Figure 9 demonstrates that the degree of enhancement of the mixture conductivity and the concentration at which the enhancement occurs are significantly influenced by the variation in the shape of the grains. The increase occurs at a critical concentration of about 33% for spherical grains (aspect ratio 1:1). The thinner the insulator discs are, the thinner the insulating layers that develop between the grains of the semi-conductor, enhancing the conductivity and enhancing the percolation concentration. As the aspect ratios changes from 9:1 to 1:1, there is are no much changes at the critical conductor concentration (from 35.4% to 33.3%). This is clear because the long aspect ratios of the grains are elongated in the stream of the current. As the aspect ratios changes from 1:1 to 0.0005:1, there is are some big changes at the critical conductor concentration (from 33.4% to 99.4%). This is because the long aspect ratios of the grains are elongated in perpendicular to the current. So, as the long aspect ratio of the insulator grains are increased, then the insulator cut the pathways between the electrodes, and accordingly it delays the formation of the percolation threshold.

Studies have indicated that the conductivity of substances is significantly influenced by the size of their grains. Furthermore, the conductivity characteristics of materials are significantly influenced by the microstructure, and texture of the mixture.

**Fig. 10:**
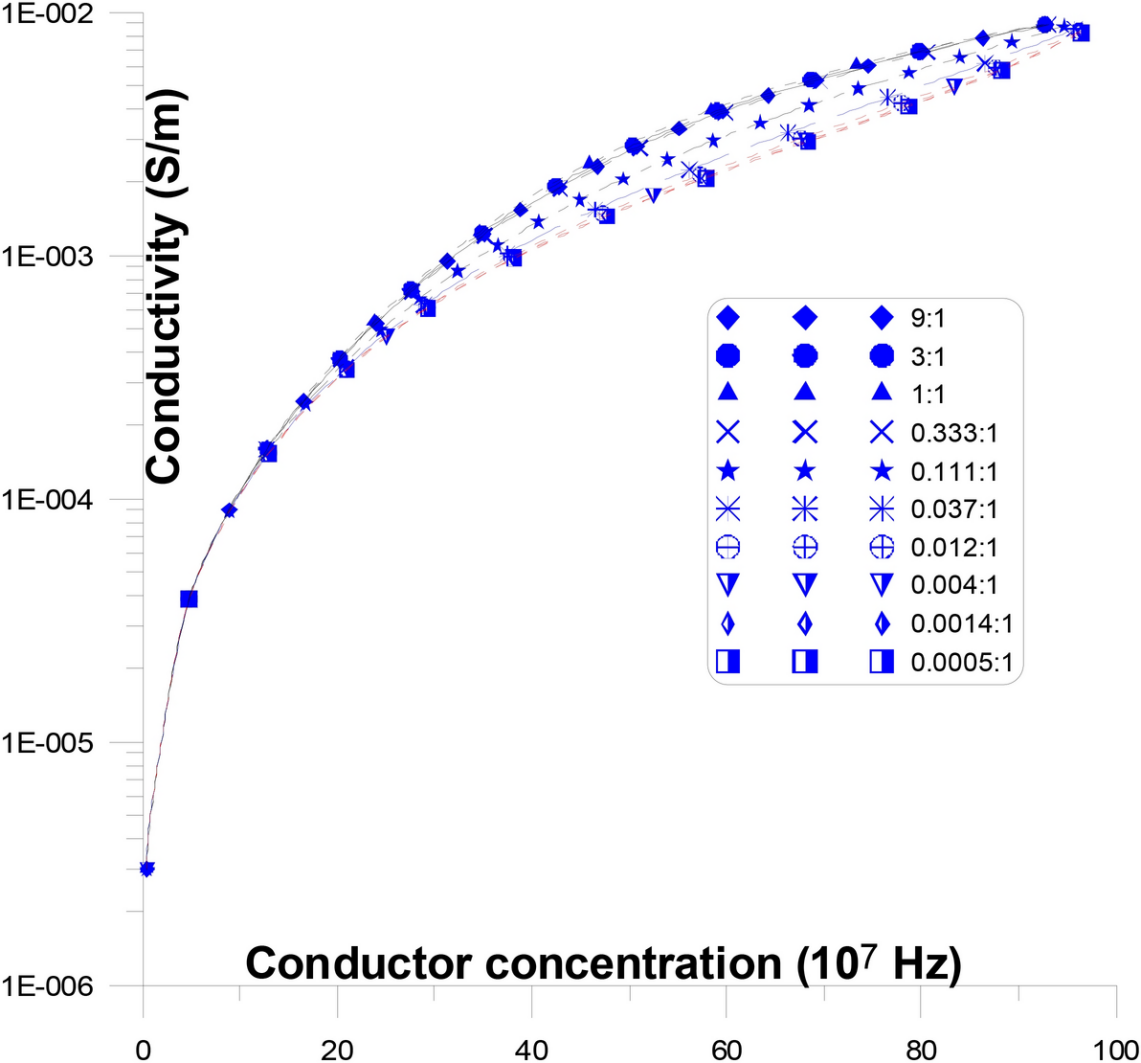
Conductivity as a function of different conductor concentration, with different insulator aspect ratio discs, perpendicular to the same direction of the current, at frequency (10^7^ Hz).

Figure 10 displays the fluctuations of the conductivity as a function of changing conductor concentration, with discs representing the different insulator aspect ratios, with the stream of current, at frequency (10^7^ Hz). At this figure, conductor discs with varying aspect ratios (from 9:1 to 0.0005:1) are present with the stream of current. When the aspect ratio is changed, the conductivity’s overall response to concentration is repeated. The conductivity gradually rises with the increase in conductor concentration from the value of the insulator conductivity (high frequency) until it reaches the conductor conductivity value, with no percolation threshold peak. It is noticed that, at low conductor concentrations, as the aspect ratios of the conductor increase (from 9:1 to 0.0005:1) the conductivity value does not change completely due to the small effect of the insulator with the stream of current, at high frequency. It is noticed that, at moderate conductor concentrations, as the aspect ratios of the conductor increase (from 9:1 to 0.0005:1) the conductivity value shows some fluctuations of increase. It is noticed that, at high conductor concentrations, as the aspect ratios of the conductor increase (from 9:1 to 0.0005:1) the conductivity value merges to the value of the conductor.

In the frame of grain shape variations, as described by EMT applied here, the theoretical results cover the experimental range of variations of the dielectric constant and conductivity at different concentrations. It can be stated that the random distribution of experimental results could be ascribed to the granular shape of the components. The peaks of dielectric constant (Fig. 3 and 5), or the abrupt increases of conductivity (Fig. 7 and 9), of each theoretical curve corresponds to the critical concentration of such curve. It reflects the grain shape effect within the sample together with the phenomenon of induced polarization. Such induced polarization is a characteristic phenomenon in such measured rock samples.

When the conducting grains stretch in the same direction as the current, there is a greater chance of a connection forming between the electrodes. When the insulating granules extend in the same direction as the current, there is a higher chance that they will impede the connection between the electrodes. When the conducting grains stretch perpendicular to the direction of the current, the likelihood of establishing a connection between the electrodes reduces. When the insulating granules elongate perpendicular to the direction of the current, there is a greater chance that they will impede the connection between the electrodes. The conducting routes are closed when the insulating grains become longer perpendicular to the direction of the current. The likelihood that conducting routes increases with the length of the conducting grains in the current stream. The conducting grains are shown to expand with the current stream and the approximate critical percolation concentration values in Table (2). An increase in conductivity results from the growth of conducting clusters or what appear to be semi-conducting clusters between grains. The primary reason for the increase in conductivity is the dispersion of particle sizes. The main factors that determine the electrical properties of granular materials are their texture, size, and surface roughness. Alterations to the grain sorting, mixing, or any other textural parameter may have an effect on the electrical properties of the samples. To sum up, the figure highlights the significance of grain form and how it affects granular materials’ conductivity and dielectric constant. When analyzing the conductivity and dielectric constant of granular materials with varying grain morphologies, EMT is a useful instrument.

**Fig. 11:**
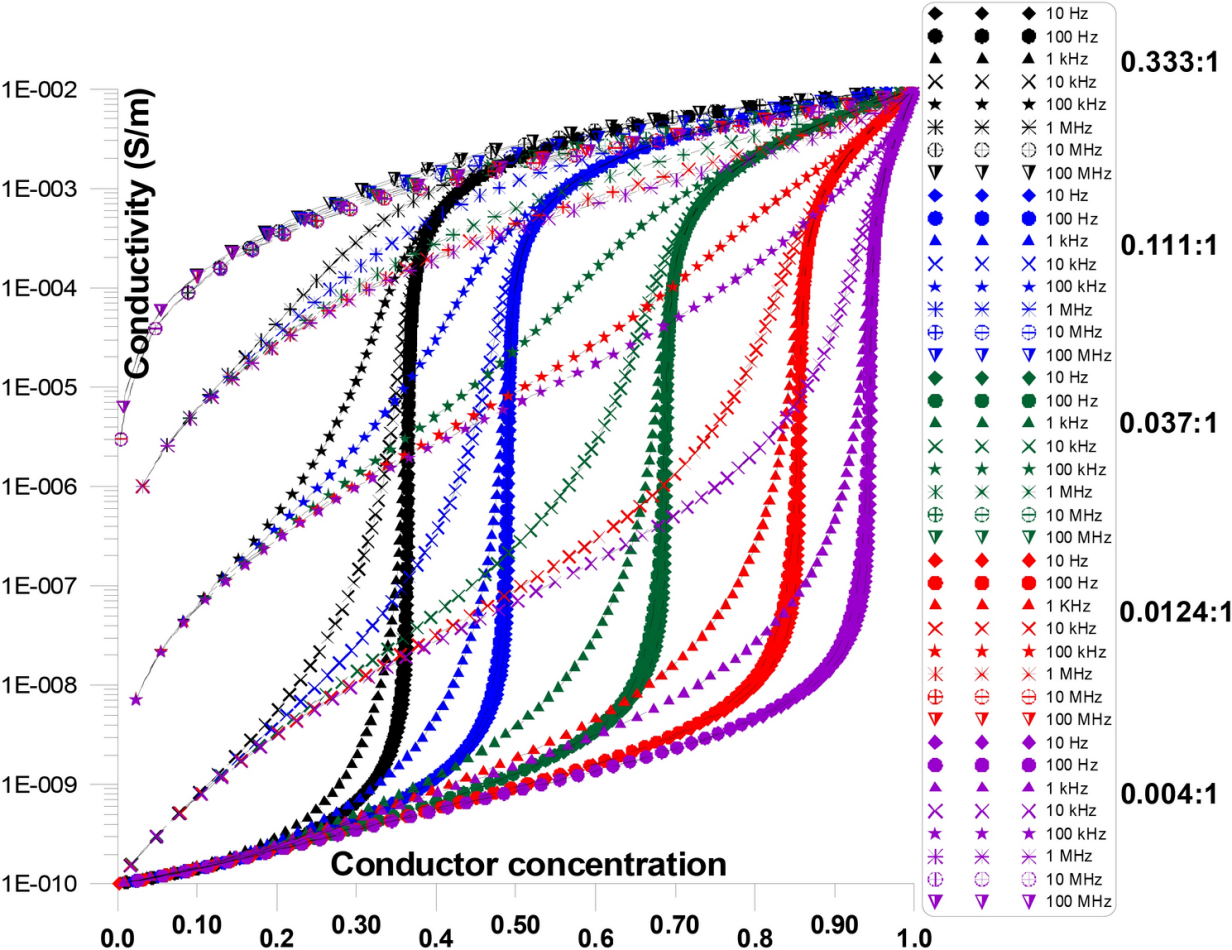
Displays the conductivity at various frequencies and grain shape aspect ratios (ranging from 0.333:1 to 0.004:1) as a function of conductor concentration.

Figure 11 displays the conductivity at various frequencies and grain shape aspect ratios (ranging from 0.333:1 to 0.004:1) as a function of conductor concentration. The figure shows that, particularly at low conductor concentrations, the initial values of conductivity develop as frequency goes up. Furthermore, the critical conductor percolation threshold increases with increasing grain shape aspect ratios (from 0.333:1 to 0.004:1).

**Fig. 12:**
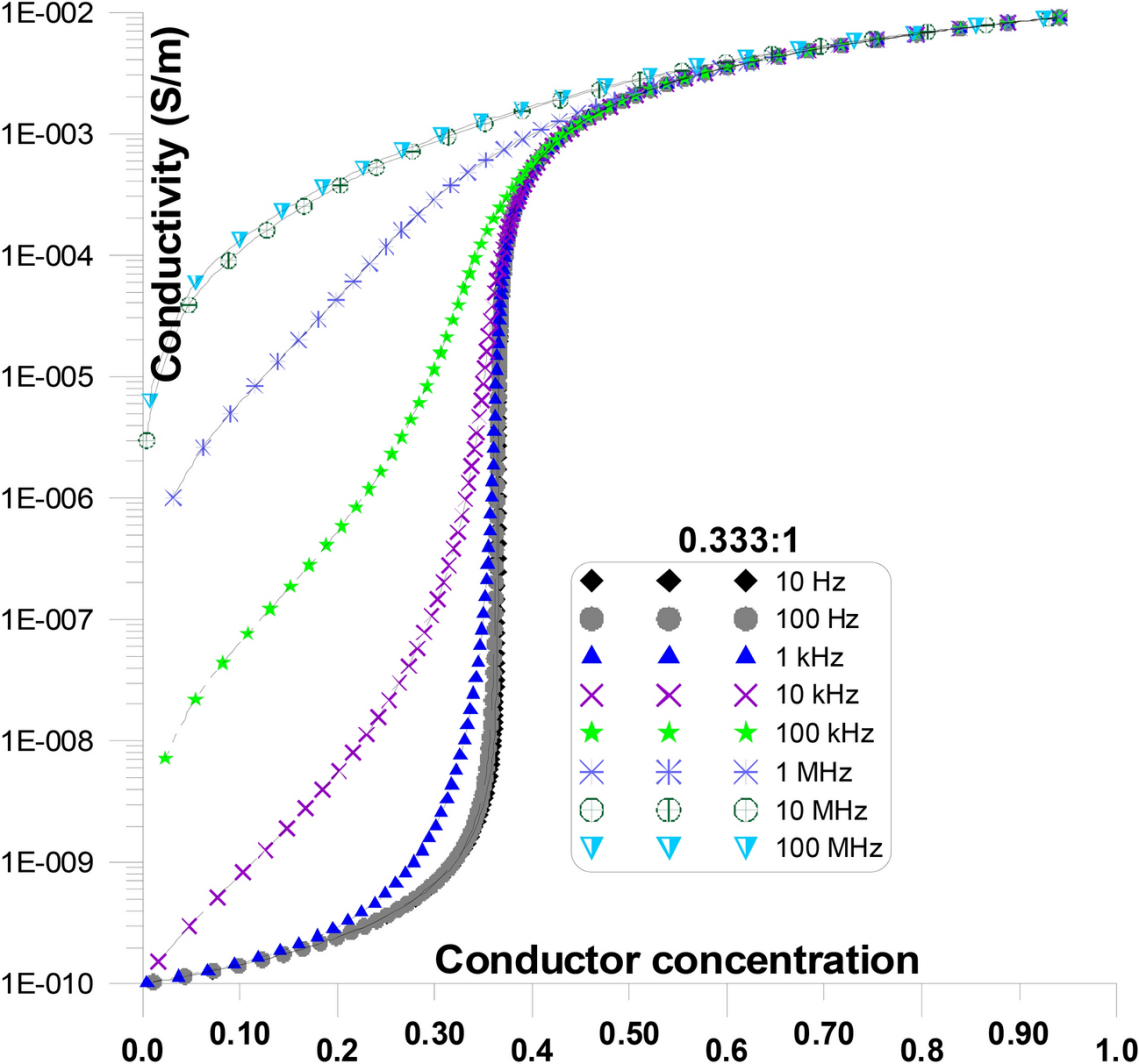
Displays the conductivity at various frequencies and grain shape aspect ratio of 0.333:1, as a function of conductor concentration.

Figure 12 displays the conductivity at various frequencies and grain shape aspect ratios of 0.333:1, as a function of conductor concentration. Figure 12 is only a part of Fig. 11 for an aspect ratio of 0.333:1. The figure shows that, particularly at low conductor concentration, the initial values of conductivity increases as frequency increases. Conductivity value at ~ 0.05 conductor concentration is in the order of 10^–10^ S/m for 10 Hz and it increases to 3X10^–10^ S/m, 10^–8^ S/m, 10^–6^ S/m, 10^–5^ S/m, for 1 kHz, 10 kHz, 100 kHz, 1 MHz, respectively. Furthermore, the sharp increase of the critical conductor percolation threshold decreases with increasing frequency ^74–78^.

**Fig. 13:**
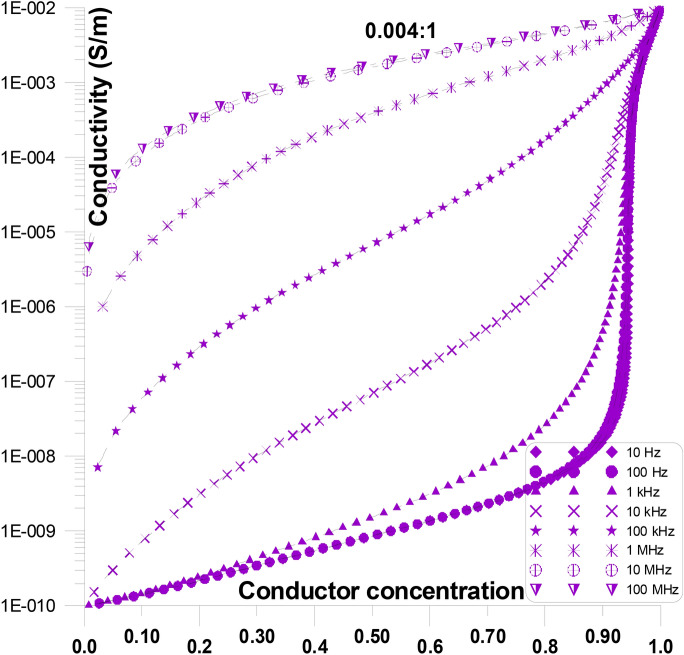
Displays the conductivity at various frequencies and grain shape aspect ratio of 0.004:1, as a function of conductor concentration.

Figure 13 displays the conductivity at various frequencies and grain shape aspect ratios of 0.004:1, as a function of conductor concentration. Figure 13 is only a part of Fig.  11 for an aspect ratio of 0.004:1. The figure shows that, particularly at low conductor concentration, the initial values of conductivity increases as frequency increases. Conductivity value at ~ 0.1 conductor concentration is in the order of 1.3X10^–10^ S/m for 10 Hz and it increases to 6X10^–10^ S/m, 4X10^–8^ S/m, 5X10^–6^ S/m, 8X10^–5^ S/m, for 10 kHz, 100 kHz, 1 MHz, 10 MHz, respectively. Moreover, when frequency increases, the crucial conductor percolation threshold’s steep rise reduces. Additionally, the conductivity values’ flat response gets significantly flatter as the conductor concentration increases.

**Fig. 14:**
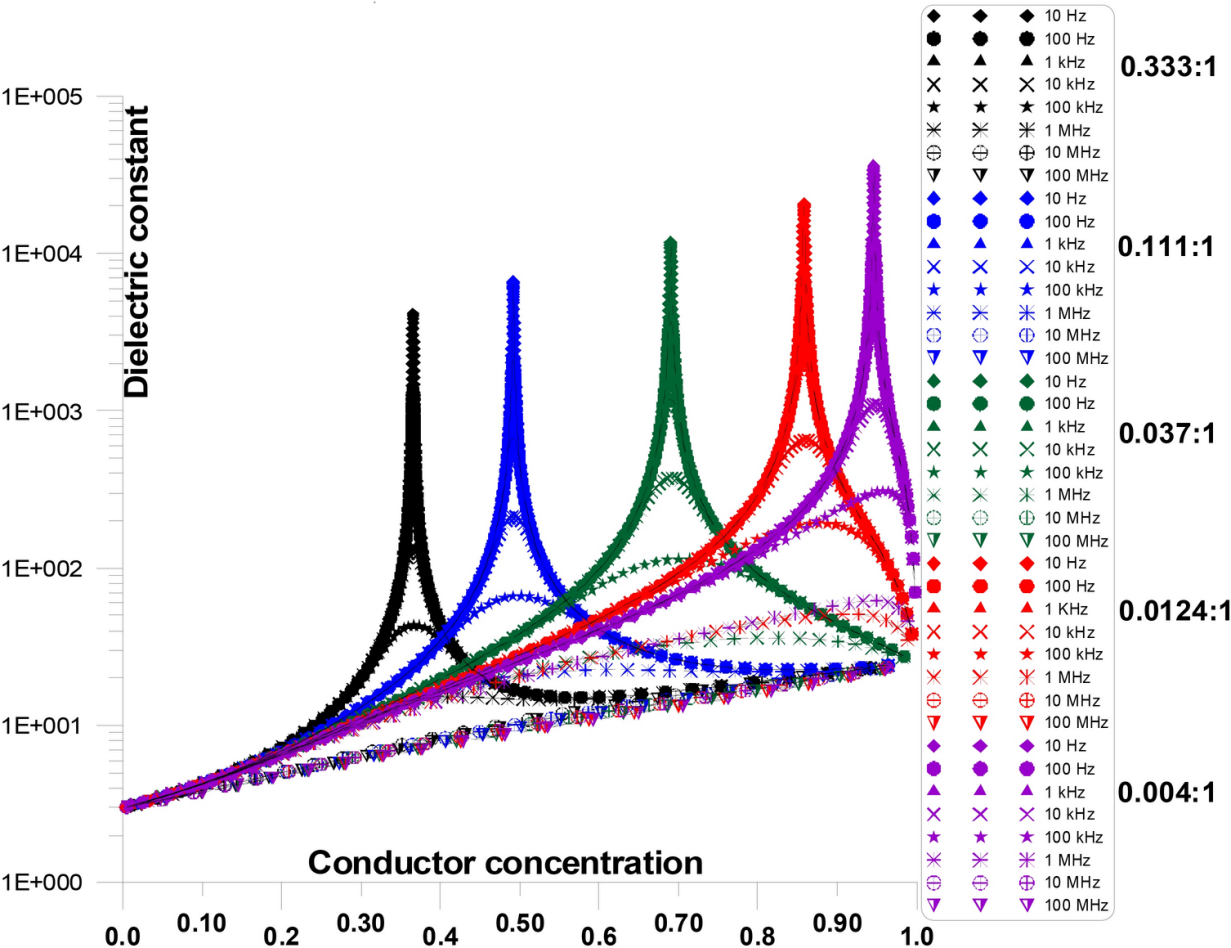
Displays the dielectric constant at various frequencies and grain shape aspect ratios (ranging from 0.333:1 to 0.004:1) as a function of conductor concentration.

Figure 14 displays the dielectric constant at various frequencies and grain shape aspect ratios (ranging from 0.333:1 to 0.004:1) as a function of conductor concentration. The figure shows that, particularly at low conductor concentrations, the initial values of dielectric constant increases as frequency goes up. Furthermore, the critical conductor percolation threshold increases with decreasing grain shape aspect ratios (from 0.333:1 to 0.004:1).

**Fig. 15:**
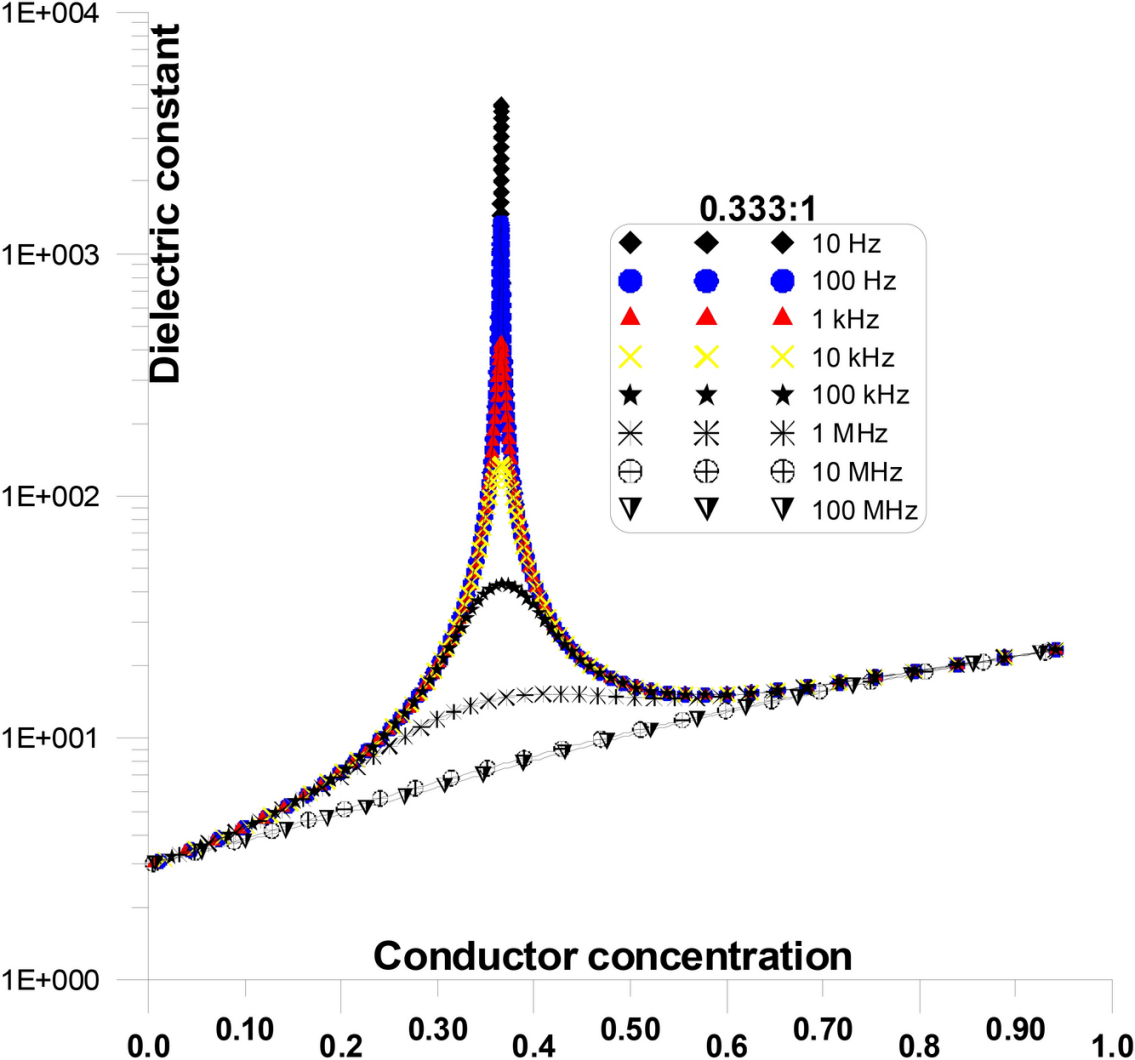
Displays the dielectric constant at various frequencies and grain shape aspect ratios of 0.333:1, as a function of conductor concentration.

Figure 15 displays the dielectric constant at various frequencies and grain shape aspect ratios of 0.333:1, as a function of conductor concentration. Figure 15 is only a part of Fig. 14 for an aspect ratio of 0.333:1. The figure shows that, particularly at critical conductor concentration, the initial values of dielectric constant decreases as frequency increases. Dielectric constant value for 10 Hz is ~ 4000 and it decreases 6 at 10 MHz.

**Fig. 16:**
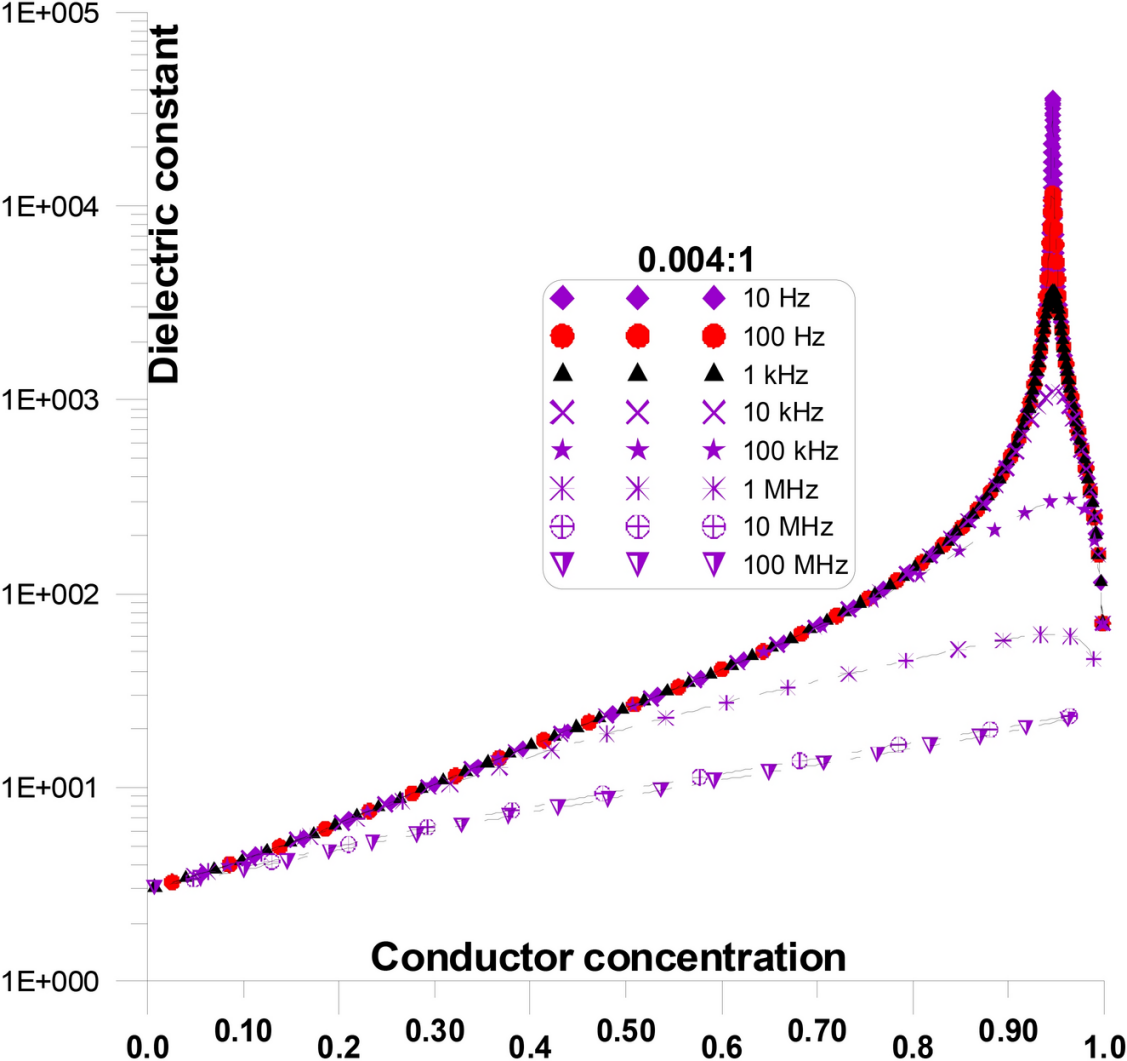
Displays the dielectric constant at various frequencies and grain shape aspect ratio of 0.004:1, as a function of conductor concentration.

Figure 16 displays the dielectric constant at various frequencies and grain shape aspect ratio of 0.004:1, as a function of conductor concentration. Figure 16 is only a part of Fig. 11 for an aspect ratio of 0.004:1. The figure shows that, particularly at critical conductor concentration, the initial values of dielectric constant decreases as frequency increases. Dielectric constant value for 10 Hz is ~ 40,000 and it decreases 10.5 at 10 MHz. Moreover, when frequency increases, the crucial conductor percolation threshold’s steep rise reduces.

## Effect of grain shape

The shape elongation of grains in mixtures can have a significant impact on the electrical characteristics of the materials. The electrical properties of mixtures vary depending on the elongation direction of the grain shape. When the grains are elongated in a certain direction, they can block potential pathways for electricity between electrodes, resulting in changes in the electrical properties of the mixture.

The correlation between grain shape elongation and electrical properties has bring in significant attention in materials science research, including the development of advanced electronic devices, energy storage systems, and sensors.

Different grain shapes provide varying pathways for the movement of electrons, affecting the overall flow of electric current within a mixture. The way in which grains interact, align, or hinder electron movement plays a vital role in determining the electrical conductivity of mixtures.

Grain boundaries are the interfaces between individual crystallites or grains in materials. These boundaries can have a significant impact on the electrical conductivity of a material, as they often act as barriers to the movement of electrons. When an electric current is applied to a material, electrons attempt to move through the material, enabling electrical conduction. However, the presence of grain boundaries can impede this process. Electrons can be scattered at these boundaries due to differences in crystal orientation and defects, leading to a reduction in overall conductivity. As the average grain size decreases, the grain boundaries per unit volume increase, leading to a higher probability of electron scattering. This results in a reduction in electrical conductivity for materials with smaller grain sizes.

Grain shape plays a crucial role in determining the electrical properties of materials, whether they are conductors or insulators. The understanding of how grain shape influences electrical behavior is of paramount importance in various fields, including electrical engineering, materials science, and electronics. In electrical engineering, grain shape can have a significant influence on the behavior and performance of mixtures.

The primary distinction between conductors and insulators lies in their electron mobility. Conductors have free electrons that can move easily, while insulators have fewer free electrons and higher resistance to electron flow. The material’s grain shape exerts an immediate impact on the change in the conductivity of electrons. Understanding the distinctions of these distinctions is crucial for comprehending how grain shape affects the electrical properties of mixtures.

Different grain shapes provide varying pathways for the movement of electrons, affecting the overall flow of electric current within a mixture. The way in which grains interact, align, or hinder electron movement plays a vital role in determining the electrical conductivity of mixtures.

As the frequency of the electrical field increases, the conductivity of conductor elongated shape of such a grain typically exhibits a stronger dependence on frequency compared to particles with a more spherical shape. This means that the conductivity of mixtures containing conductor grains can be more sensitive to changes in frequency. Not only does grain shape impact conductivity, but it also influences the dielectric properties of mixtures. The shape of the grains can alter the dielectric response of the mixture at different frequencies. For example, insulator grains with irregular shapes can lead to variations in the dielectric constant of the mixture as the frequency changes. This means that the ability of the mixture to store electrical energy, or its capacitance, can be affected by the shape of the grains.

In mixtures with irregularly shaped grains, electrons may encounter more barriers and obstacles due to the rough surfaces ^79^. This can lead to increased scattering and a higher resistance to electron flow ^80,81^. On the other hand, mixtures with more spherical grains provide smoother pathways for electrons, reducing scattering and improving conductivity. So, grain shape can directly influence the electron transport mechanisms, affecting the overall electrical properties of the mixture.

Grain shape affects the conductivity of both conductors and insulators. In conductors, grain shape influences the movement of free electrons, while in insulators; it affects the flow of charge carriers within the material.

Highly elongated conductor grains tend to exhibit higher conductivity due to enhanced grain boundary connectivity. As grains elongate along one particular direction, the resulting larger contact surface area promotes electron flow through the grain boundaries, reducing resistance and facilitating electron transfer in the material. Along with increased electrical conductivity, conductor grain shape elongation also reduces the resistivity of the material. The elongated grain structure mitigates the scattering of charge carriers, reducing their collision frequency and consequently lowering resistivity. Additionally, grain shape elongation has been observed to positively impact the charge carrier mobility, enhancing the material’s ability to transport charges efficiently.

Grain shape elongation significantly influences the dielectric properties of materials. Elongated grains aligning in specific directions can lead to anisotropic behavior in dielectric constant and dielectric loss. The alignment of elongated grains can induce preferential polarization in ferroelectric materials, resulting in improved electric response.

When conductive grains are elongated, it creates a greater path for electrical current to flow through. This increased contact area between grains allows for more efficient conduction of electricity or electrical charges. On the other hand, insulator grains with elongated shapes tend to hinder the flow of electrical current, reducing their conductivity.

The elongated shape (grain clustering) allows for more efficient electron transfer between adjacent grains, resulting in lowering resistance and increased conductivity. As the elongation of insulator grains increases, their ability to insulate electric current decreases.

Surface roughness contributes to the electrical properties of grains. A smoother surface allows for better insulation, whereas a rough surface can create leak paths for the current. Understanding the factors that influence the elongation of insulator grains is crucial in evaluating their electrical behavior. Irregularly shaped particles with jagged edges tend to elongate more compared to spherical particles.

## Conclusion

Mixtures can drastically change the electrical properties of samples due to their vast variety of electrical behaviors. Furthermore, there is a lot of interest in how grain shape anisotropies affect the electrical and dielectric properties of rocks. The frequency, texture, concentrations, particle size, shape, and other factors all affect these characteristics. EMT can explain the abnormal electrical characteristics of mixtures in a unique way. The impact of concentration and grain shape was the main focus of this investigation. Grain shape has been shown to be an effective parameter influencing the electrical properties of the sample. Using EMT and grain shape alterations used here, it was possible to cover the experimental range of changes of the dielectric constant and conductivity at different concentrations. Generally speaking, when particle shape decreases, conductivity and dielectric constant change extremely. Conductivity increases with more conducting clusters between grains, whereas dielectric constant increases with decreased porosity across particle sizes. Grain shape affects the electrical properties of the materials, sometimes more so than conductor concentrations. Based on the electrical characteristics of the curves, the conductivity and dielectric constant change due to the formation of conducting clusters, between grains or clusters. Conductivities are then altered by employing spherical, oblate, and prolate ellipsoidal shape parameters within a broad effective medium. Grain boundaries have the ability to impede the flow of electrical current, which in turn reduces overall conductivity due to electron scattering. Understanding how the elongation of conductive and insulator grains influences electrical behavior can provide information about the theoretical and practical aspects of understanding and forecasting the electrical characteristics of the mixtures while dealing with anomalous behavior.

## Data Availability

"The datasets used and/or analysed during the current study available from the corresponding author on reasonable request".

## Third party material

All of the material is owned by the authors and/or no permissions are required.
